# Cerebellar granule neurons induce Cyclin D1 before the onset of motor symptoms in Huntington’s disease mice

**DOI:** 10.1186/s40478-022-01500-x

**Published:** 2023-01-20

**Authors:** Susanne Bauer, Chwen-Yu Chen, Maria Jonson, Lech Kaczmarczyk, Srivathsa Subramanya Magadi, Walker S. Jackson

**Affiliations:** 1 Wallenberg Center for Molecular Medicine, Department of Biomedical and Clinical Sciences, Linköping University, Room 463.10.30, Linköping, Sweden; 2 German Center for Neurodegenerative Diseases, Bonn, Germany

## Abstract

Although Huntington’s disease (HD) is classically defined by the selective vulnerability of striatal projection neurons, there is increasing evidence that cerebellar degeneration modulates clinical symptoms. However, little is known about cell type-specific responses of cerebellar neurons in HD. To dissect early disease mechanisms in the cerebellum and cerebrum, we analyzed translatomes of neuronal cell types from both regions in a new HD mouse model. For this, HdhQ200 knock-in mice were backcrossed with the calm 129S4 strain, to constrain experimental noise caused by variable hyperactivity of mice in a C57BL/6 background. Behavioral and neuropathological characterization showed that these S4-HdhQ200 mice had very mild behavioral abnormalities starting around 12 months of age that remained mild up to 18 months. By 9 months, we observed abundant Huntingtin-positive neuronal intranuclear inclusions (NIIs) in the striatum and cerebellum. The translatome analysis of GABAergic cells of the cerebrum further confirmed changes typical of HD-induced striatal pathology. Surprisingly, we observed the strongest response with 626 differentially expressed genes in glutamatergic neurons of the cerebellum, a population consisting primarily of granule cells, commonly considered disease resistant. Our findings suggest vesicular fusion and exocytosis, as well as differentiation-related pathways are affected in these neurons. Furthermore, increased expression of cyclin D1 (*Ccnd1*) in the granular layer and upregulated expression of polycomb group complex protein genes and cell cycle regulators *Cbx2, Cbx4* and *Cbx8* point to a putative role of aberrant cell cycle regulation in cerebellar granule cells in early disease.

**Supplementary Information:**

The online version contains supplementary material available at 10.1186/s40478-022-01500-x.

## Introduction

Huntington’s disease (HD) [OMIM:143100] is a progressive and fatal neurodegenerative disease caused by a CAG repeat expansion in exon 1 of the Huntingtin gene (*HTT*), which results in an expanded polyglutamine stretch in the Huntingtin protein (HTT). While both wild-type and mutant HTT (mHTT) are expressed ubiquitously, the striatum is the region most affected in HD. In particular, GABAergic striatal projection neurons (SPNs) show pronounced selective vulnerability, with early degeneration of dopamine 2 receptor (Drd2)-expressing SPNs of the indirect pathway, followed by Drd1-expressing SPNs of the direct pathway [1]. In addition to striatal degeneration, renewed attention has been placed on cerebellar pathology and reassessment of its potential role in clinical symptoms of HD.

The cerebellum plays a role in functions commonly affected in HD patients, such as impairment of dexterity, postural instability, or ataxia-like symptoms. Indeed, the presence of neuropathology in the cerebellum has been described in HD patients and mouse models and is predominantly characterized by Purkinje cell loss, while the granular layer is relatively spared [2–5]. Neuroimaging showed degeneration of cerebellar gray matter in anterior and posterior lobules of the cerebellum in patients with mild motor symptoms [6]. Similarly, significant loss of Purkinje neurons was found in patients with motor phenotypes specifically, corroborating the involvement of the cerebellum in HD symptoms and a correlation of HD phenotype with cerebellar pathology [4]. Interestingly, there is increased functional connectivity between the striatum and cerebellum in asymptomatic children carrying CAG expansions, which declines in an age- and CAG repeat length-dependent manner [7]. Thus, the cerebellum may help to maintain normal motor function in early HD, compensating for the loss of striatal function [8]. Consequently, the spectrum of symptoms in HD may be influenced by loss of cerebellar compensation in addition to striatal dysfunction, making the cerebellum an interesting therapeutic target in HD.

Despite the importance of cerebellar disturbances in HD, the mechanisms behind them remain largely unclear. For example, the occurrence and degree of cerebellar pathology do not strictly correlate with CAG repeat length or the degree of striatal degeneration [2, 4, 9], which begs the question of what other mechanisms drive cerebellar degeneration in HD. Previous studies of mouse and human samples reported gene expression changes in the cerebellum, though delayed in severity compared to the striatum [10–12]. In general, functional pathways changed in the cerebellum were similar to those in the striatum and other brain regions [12, 13], suggesting differences in timing and severity of change may be the result of different inherent capacities of these regions to respond to mHTT [12]. Although recent studies of human and mouse striata identified cell type-specific gene expression responses to HD [14], similar studies of cerebella are missing. Since a differential vulnerability between cerebellar cell types has been reported, we studied responses of excitatory (vGluT2^+^) and inhibitory (Gad2^+^) cerebellar populations using cell type-specific translatome analysis with RiboTag [15] during an early, pre-symptomatic disease stage in S4-HdhQ200 mice. Surprisingly, cerebellar vGluT2^+^ neurons showed the strongest response with 626 differentially expressed genes (DEGs) which were enriched for vesicular exocytosis and included upregulation of cell cycle regulators Cyclin D1 and chromobox (Cbx) protein genes. This study suggests granule cells, commonly considered resistant in HD, may be affected early in disease.

## Methods

Ethical permissions for this work were granted by the Linköpings djurförsöksetiska nämnd (permission # 14741-2019) and the Landesamt für Natur, Umwelt und Verbraucherschutz Nordrhein-Westfalen (permission #s 84-02.04.2013.A169 and 84-02.04.2013.A128).

All HD mice were heterozygous as the very long mutation may partially inactivate the gene’s native function, and homozygotes may partially develop *Htt* knock-out phenotypes*.* Additional details are provided in supplemental methods.

### Mouse breeding

#### For RiboTag and histology experiments

S4-HdhQ200 mice were crossed three times to homozygous RiboTag mice (B6N.129-Rpl22^tm1.1Psam^/J, line #011029; Jackson Laboratory, Bar Harbor, ME), on a 129S4 background (> 99.5% 129S4) to establish Hdh^Q7/Q200^/Rpl22-HA^flox/flox^ mice. Cre-driver lines vGluT2-IRES-Cre [16] (Slc17a6^tm2(cre)Lowl^/J, line #016963; Jackson Laboratory), Gad2-IRES-Cre [17] (Gad2^tm2(cre)Zjh^/J, line #010802; Jackson Laboratory), and PV-IRES-Cre [18] (B6;129P2-Pvalb^tm1(cre)Arbr^/J, line #008069; Jackson Laboratory), were each backcrossed to be > 99% 129S4 [19]. To obtain experimental animals, male Hdh^Q7/Q200^/Rpl22-HA^flox/flox^ mice were crossed with female Hdh^Q7/Q7^ mice homozygous for Cre, producing offspring that are heterozygous for Cre and RiboTag, and either heterozygous or wild-type for *Htt*. Mice were sacrificed at approximately 9 months (average: 41.5 weeks, SD: 1.8; range: 39–45 weeks) by CO_2_ asphyxiation. The olfactory bulb was removed from the second hemisphere and the cerebellum and cerebrum were separated, flash-frozen on dry ice, and stored at − 80 to − 72 °C. Details are also provided in Supplementary Figure 1. The most abundant CAG repeat lengths were measured for each sample by fragment analysis. The smallest, median, and longest CAG repeat in the sample population: vGluT2 = 203, 205, 209; Gad2 = 201, 203, 207; PV = 202, 204, 207. For behavioral experiments, HdhQ200 mice were bred to 129S4 to generation 12 and subsequently bred to 129S4 mice carrying the Disrupted in schizophrenia 1 (*Disc1*) and non-agouti genes from C57Bl/6NTac mice that were 99.8% 129S4. The smallest, median, and longest repeat in the sample population were 160, 172, and 175 CAG triplets, respectively.

### Behavior

All tests were performed on the same cohort of control (3 male, 4 female) and HD mice (7 male, 5 female) between 3 and 18 months. Following 2 initial training days, motor tests were performed every 2–3 months and animals were weighed monthly. Mice were tested on the accelerating rotarod (ENV-574M, Mead Associates Inc, Fairfax, VT) for 300 s, with acceleration every 30 s from 4 revolutions per minute (rpm) to 40 rpm, and latency to fall was recorded. Followed by the balancing beam paradigm, in which mice crossed an ascending, narrow beam (1 m, 17° ascent, and 1.5 − 0.5 cm taper across the width) to reach a black box at the high end and time needed to traverse the beam was recorded. At each time point, mice performed two repeat balancing tasks, interspersed by burrowing. Burrowing behavior of animals was tested for 1 h in the light phase. Individual mice were placed in a cage with 200 ml of standard bedding substrate and a burrow made of a 20 cm long black plastic tube with a diameter of 7 cm, with the open end raised 3 cm and the closed end raised 1 cm from the cage floor. Removed bedding material was used as a measure of burrowing activity.

### Neuropathology

#### Tissue preparation

Mice were sacrificed by CO_2_ asphyxiation followed by transcardial perfusion with 10% formalin solution. Dissected brains were separated into hemispheres along the midline and fixed for 2 days at 4 °C with gentle shaking. Fixed brains were paraffinized with each cassette containing age-matched HD and control samples and cut into 4 µm sections.

#### Immunohistochemistry/immunofluorescence staining

Tissue sections were de-paraffinized and boiled in citrate buffer (pH 8) for 20 min for antigen retrieval, then cooled for 20 min at room temperature (RT). For NII staining, sections were incubated with 25 μg/ml proteinase K at 30 min/37 °C to remove cytosolic HTT. Sections were treated with 0.3% H_2_O_2_ for 30 min/RT, then permeabilized with blocking buffer (PBS with 2.5% normal horse serum (NHS)) for 1 h/RT, followed by incubation with primary antibody for 30 min/RT. Sections were washed twice in PBS for 5 min and secondary antibody was incubated for 30 min/RT, followed by 2 × 5 min PBS washes. For immunohistochemistry, sections were colorized using NovaRED (SK-4800, Vector Laboratories, Burlingame, CA). Primary antibodies: Huntingtin 1:100 (ab109115, Abcam, Cambridge, UK); GFAP 1:500 (GA524, Dako Omnis); Iba1 1:200 (019-10741, Wako Chemicals). Fluorescent secondary antibodies: Alexa Fluor 488 donkey anti-rabbit 1:250 (Jackson ImmunoReseach, AB_2313584).

In situ hybridization (ISH) was performed according to the RNAScope Fast-RED protocol followed by autofluorescence quenching for 5 min with TrueView (Vector Laboratories). Counterstaining with DAPI (1:20,000 in PBS with 2.5% NHS) was performed to identify Htt aggregates that are NIIs.

#### Image analysis and quantification

For *Htt* ISH, images of sections were taken with a Zeiss Axio A1/D1 microscope (Zeiss, Oberkochen, Germany) and deconvoluted using Huygens software (Scientific Volume Imaging (SVI), Hilversum, Netherlands). For quantitative analysis of Htt aggregates, *Htt* RNA staining, and HTT/DAPI colocalization, particle counting was done using IMARIS with default settings (Bitplane, Oxford Instrument, England). For *Ppp1r1b* ISH integrated density was measured in FIJI. *Ccnd1* ISH quantification was performed on matched HD and control sections from the same cassette, and imaged using a Zeiss LSM700 confocal microscope with identical settings. For quantification, a sum projection of Z-stacks was performed in FIJI and mean fluorescence intensity was measured in the granular layer right and left of arbor vita after background correction.

### RiboTag RNAseq

#### Preparation of RNA samples

For RiboTag Immunorepecipitation (IP) samples, cell type-specific mRNA was immunoprecipitated from brain tissue homogenates of flash-frozen cerebellum or cerebrum hemispheres, as described here [20]. In brief, homogenate was precleared by centrifugation and incubation with IgG isotype antibody-bound protein-G dynabeads (PGDB; 1009D, Invitrogen, Waltham MA). Hemagglutinin (HA)-tagged ribosomes with bound mRNAs were captured by incubation with 12CA5 anti-HA antibody (lot: 11666606001, Roche) 90 min/4 °C, followed by incubation with PGDBs for 60 min/4 °C. Beads were washed using buffers with increasing salt concentration and mRNA was eluted by phenol–chloroform extraction, followed by clean-up using the Qiagen RNeasy Mini kit. Before performing the RiboTag protocol, total RNA was isolated from tissue homogenates as input control.

#### Sequencing and alignment

Illumina TruSeq Stranded mRNA libraries were sequenced (paired-end, 100 bp) on a NovaSeq6000 (Illumina, San Diego CA) on an S4 flow cell. Library preparation and sequencing were performed at NGI Uppsala, Sweden. Alignment and mapping were performed with STAR/Salmon. Samples were kept if they contained > 24 M uniquely mapped reads.

### Bioinformatic analysis

For principal component analysis (PCA) the variance for protein-coding genes was calculated based on log-scaled transcripts per million (TPM) values across RiboTag IP or total RNA samples. The top 500 most variable genes were used for PCA. To analyze the enrichment of cell type-specific marker genes in RiboTag IP samples, we normalized log2-transformed TPM values to total RNA and calculated the row-wise z-score: Z = (x – mean(total RNA))/SD(row), where x is the sample value and SD is the row-wise standard deviation. Heatmaps were visualized with pheatmap (v1.0.12).

Differential expression analysis was performed with DESeq2 (v1.30.1) comparing disease and control samples for each cell type. Only protein-coding genes with a row-wise mean count > 10 were considered. Genes with a false discovery rate (FDR)-adjusted *p*-value ≤ 0.05 were considered as differentially expressed.

Overrepresentation analysis (ORA) for differentially expressed genes (DEGs) was performed for each cell type using clusterProfiler (v3.18.1), with a cutoff of FDR ≤ 0.05.

Gene set enrichment analysis (GSEA) for gene ontology (GO) Biological Process (c5.go.bp.v7.4.symbols; gsea-msigdb.org) and KEGG pathways (KEGG_mouse_2019; maayanlab.cloud/Enrichr) was performed with piano (v2.6.0) [21] using six methods (“mean”, “median”, “sum”, “stouffer”, “reporter” or “tailStrength”) to calculate statistical significance. Median consensus scores were calculated based on adjusted *p*-values using the integrated consensusScores function. Terms with distinct directional FDR ≤ 0.05 in at least two of the six applied gene set statistics were included. For visualization, GO terms were collapsed to parent terms by semantic similarity (Resnik, threshold = 0.8) using rrvigo (v1.2.0).

Chip-X enrichment analysis (ChEA) [22] was performed using the enrichr R-package (v3.0) to analyze the overrepresentation of DEGs among gene sets in the ChEA 2016 collection of transcription factor-associated genes.

Overlap analysis of cell type-specific DEGs from RiboTag IPs with bulk RNAseq data was calculated using Fisher’s exact test with the GeneOverlap R package (v1.26.0), using the average number of protein-coding genes detected in IP samples (12,519) as background. External data sets: HD [GEO:GSE65776]; SCA1 [GEO:GSE122099].

## Results

Although conventional transgenic mice can cause severe and early disease onset, accelerating experimentation, such models are vulnerable to expression pattern artifacts [23]. We therefore employed a knock-in mouse model. The mouse model carrying a repeat of approximately 200 CAGs in the endogenous gene (HdhQ200) was initially established in a C57BL/6J background [24, 25]. Since approximately 20% of mice in this background demonstrate nocturnal hyperactivity [26], which could create gene expression noise, we backcrossed the mutant allele into the 129S4 background. To characterize the disease phenotype in this new genetic background, we performed behavioral and histological analyses on S4-HdhQ200 heterozygous mice and littermate controls (hereafter HD and control respectively) at different ages (Fig. S1).

### HD mice in the S4 background are mildly affected

**Fig. 1:**
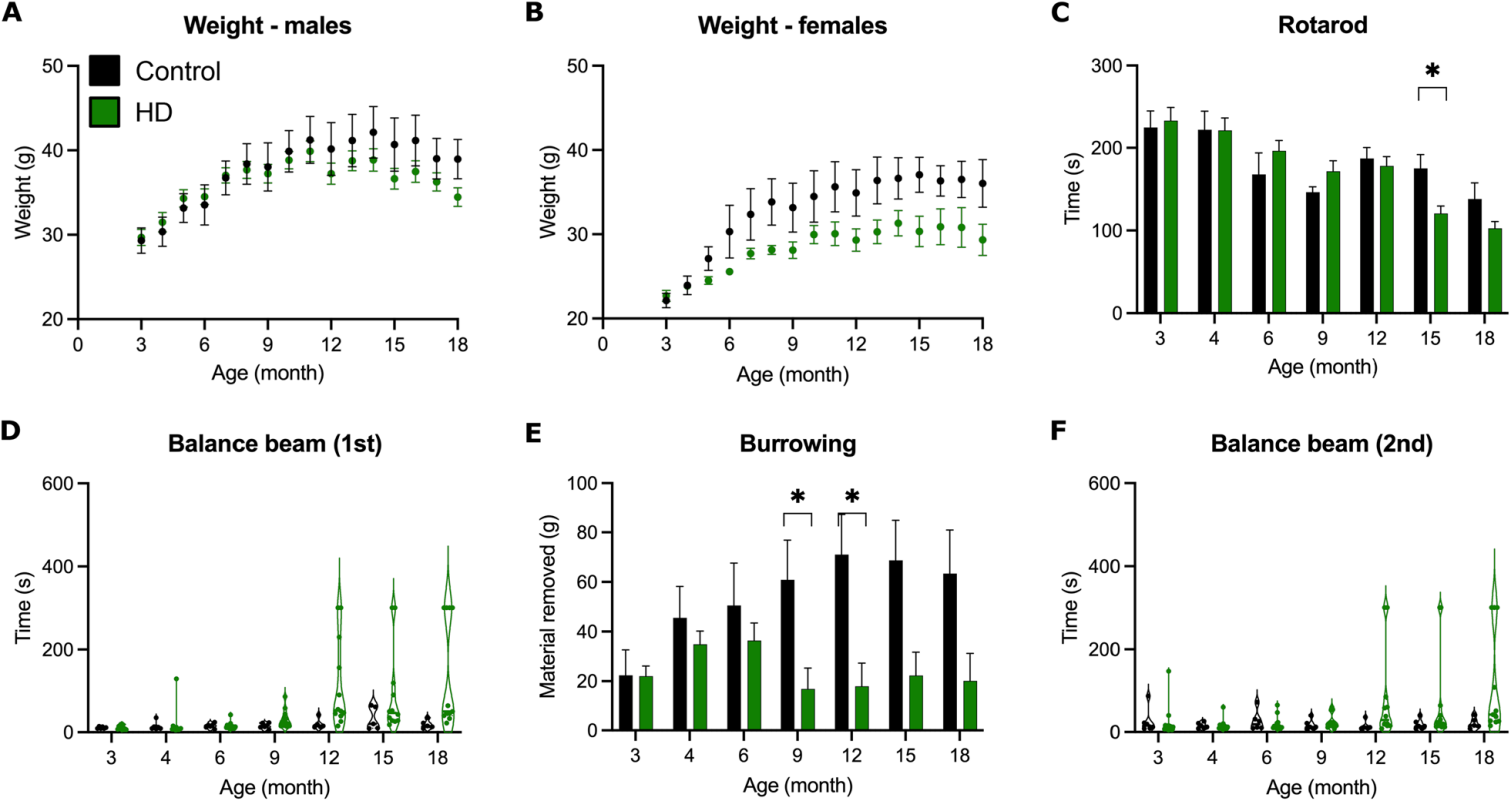
S4-HdhQ200 mice have a mild behavioral phenotype. **A**, **B** Body weight is not significantly reduced in male (**A**), or female (**B**) HD mice compared to littermate controls. **C** HD mice remain on the rotarod for less time than control mice at 15 months. **D**, **F** HD mice show no significantly different performance in the balance beam task. For each time point, two repeat measures were performed, with a one-hour burrowing period between the first (**D**) and second (**F**) test. **E** HD mice burrow less than littermate controls at 9 months and 12 months. HD n = 12 (green), control n = 7 (black), unpaired t-test without corrections for multiple testing, (*p < 0.05)

To assess behavioral changes during disease progression, we evaluated animals between the ages of 3–18 months using standard paradigms. At each experimental time point, mice performed a session on the accelerating rotarod, followed by walking on a balance beam, 1 h of burrowing and a second balance beam task. Body weight was monitored over the course of the behavioral assessment. Neither males nor females showed a significant difference in body weight between HD and controls (Fig. 1A, B), although female HD mice tended to weigh less. Rotarod performance declined with aging for both control and HD mice, but significant differences were only observed at 15 months (*p* < 0.05; Fig. 1C). Some HD mice performed very poorly on the balance beam task beginning at 12 months, but the majority performed as well as controls and the group average was similar to controls (Fig. 1D, F). Burrowing is an innate, instinctive, and rewarding behavior in mice, and burrowing paradigms are used as an assessment of overall well-being and the effects of disease on the performance of spontaneous behavior [27]. HD mice burrowed less at 9 and 12 months (*p* < 0.05). This trend continued at 15 and 18 months, but since several HD mice performed as well as controls, the differences were not significant (Fig. 1E). A larger cohort of mice may have produced more statistically significant differences, but it would not have changed the overall conclusion that S4-HdhQ200 mice showed only a very mild behavioral phenotype, even at an advanced age, similar to when this mutation is in the C57Bl/6 background [24, 25].

**Fig. 2:**
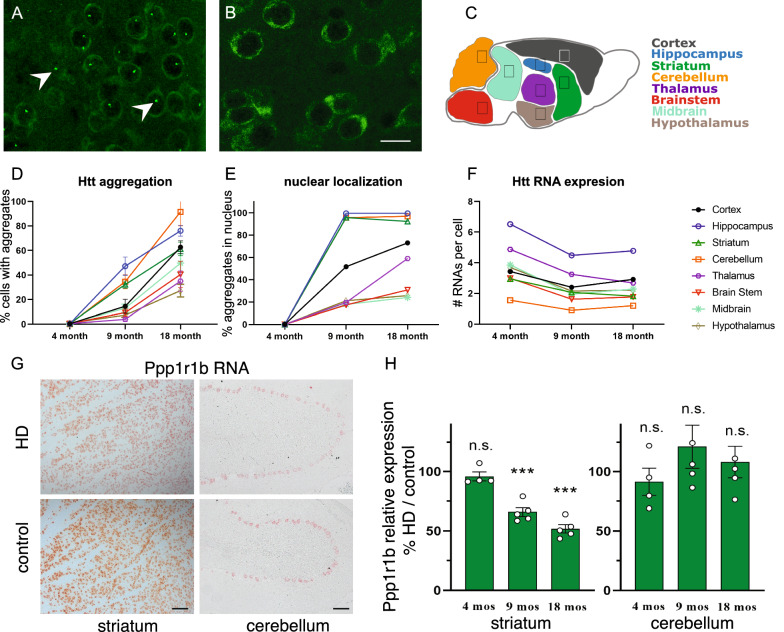
S4-HdhQ200 mice show HD-typical neuropathology by 9 months of age. **A** Htt aggregates (2 examples marked with arrowheads) present in 9 months (mos) old HD striatum (**A**), were absent from 9 mos old control striatum (**B**). **C** Overview of brain regions analyzed. **D**–**F** The proportion of aggregates per cell (**D**), the percent of aggregates that are NIIs (**E**), and the number of RNA molecules detected per cell (**F**). All charts share the key in (**F**), using the same color scheme as in (**C**); statistical analyses are presented in Fig. S2. **G**–**H** Representative images of RNA ISH for Ppp1r1b in 9 mos old brain sections (**G**), and quantification and analysis in (**H**). Unpaired t-test without corrections for multiple testing (***p < 0.001). The scale bar in (**B**) represents 25 µm, and the scale bars in (**G**) represent 100 µm

In contrast to the mild behavioral phenotype, the histopathological phenotype revealed changes consistent with HD. NIIs are common in polyglutamine diseases, such as HD and several spinocerebellar ataxias, and their extent can be indicative of disease stage. To improve detection of Htt protein aggregates, soluble Htt was partially removed by applying a mild proteinase K treatment, resulting in bright puncta on a background of diffuse staining, only in HD mice (Fig. 2A, B). The proportion of cells with proteinase K-resistant Htt aggregates was measured at 4, 9 and 18 months in eight brain regions (Fig. 2C). By 9 months of age, aggregates were detected in all brain regions, however hippocampus, striatum, and cerebellum had the highest percentage (Fig. 2D, Fig. S2). Immunofluorescent co-staining of Htt with the nuclear stain DAPI revealed that nearly all aggregates in these brain regions were NIIs (Fig. 2E, Fig S2). In contrast, aggregates in the thalamus, brain stem, midbrain, and hypothalamus were much less frequent and presented mostly as cytosolic aggregation foci, appearing like NIIs but not within a nucleus (Fig. 2D, E; Fig. S2). To determine if regions with high NII load expressed *Htt* the highest, in situ hybridization (ISH) for *Htt* mRNA was performed. Surprisingly, the striatum and cerebellum were among the lowest expressing regions, whereas the thalamus was the second highest expressing region, indicating something other than expression levels determines NII load (Fig. 2F). We also observed a significant, age-dependent downregulation of *Htt* mRNA levels in HD mice in all brain regions except the hypothalamus by 9 months (Fig. 2F; Fig. S2).

Previous analyses of similar HD knock-in mouse models revealed downregulation of common HD-associated genes in striatal cell types early in the disease [10, 14], suggesting loss of striatal identity may be one of the earliest features of HD pathology. We therefore performed ISH staining for one such marker, Ppp1r1b (DARPP32), which is abundant throughout the striatum and in Purkinje neurons of the cerebellum. Ppp1r1b was reduced by 38% in the striatum at 9 months, and 51% by 18 months but was unchanged in the cerebellum at all time points (Fig. 2G, H). Staining for Gfap and Iba1 showed no obvious signs of astrogliosis or microgliosis at any disease stage (Fig. S3). Finally, no differences were detected between HD and control mice at 4 months of age, indicating the abnormalities detected at 9 and 18 months were not caused by a developmental defect. In summary, like other HD knock-in mice, S4-HdhQ200 mice have very mild behavioral and neuropathological changes, with abundant NII accumulation in specific brain regions and Ppp1r1b reduction in the striatum at 9 months. Therefore, here we considered 9 months as an ideal early disease stage for assessment of cell type-specific translatome changes.

### Capture of cell type-specific translatomes

**Fig. 3:**
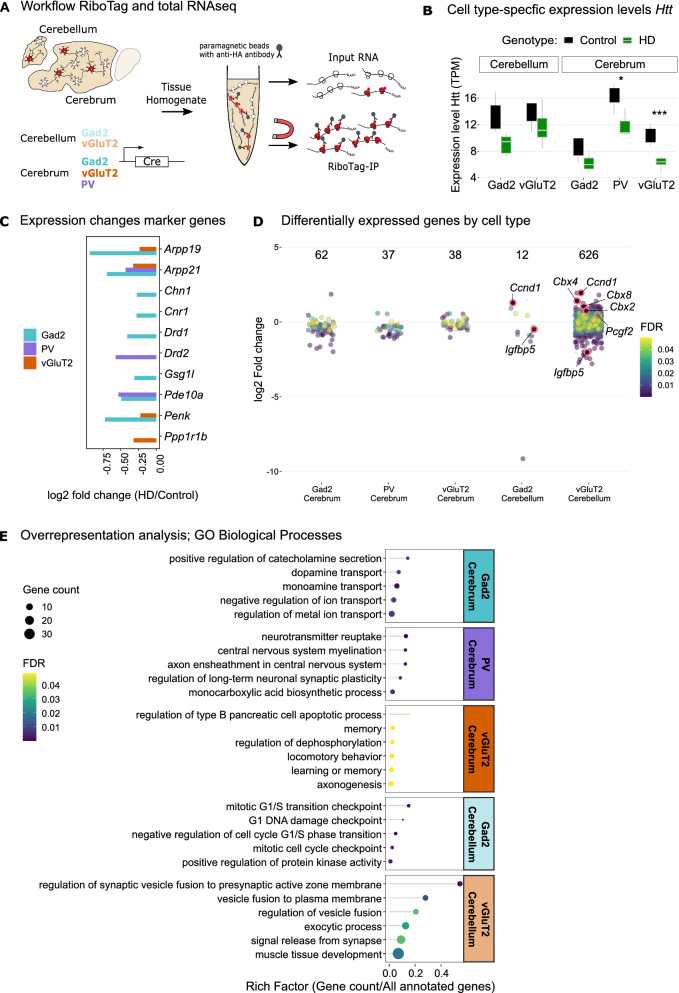
Translatome analysis with RiboTag reveals cell type-specific responses in HD. **A** Hdh^Q7/Q200^ mice homozygous for RiboTag were crossed with homozygous Cre-driver lines to facilitate cell type-specific expression of Rpl22-HA in glutamatergic (vGluT2^+^) and GABAergic (Gad2^+^) neurons in the cerebellum and cerebrum, and parvalbumin (PV^+^)-expressing neurons in the cerebrum. Cerebellum and cerebrum (without olfactory bulb) were separated and flash frozen at 9 months. For RiboTag IPs, cell type-specific HA-tagged ribosomes were immunoprecipitated from tissue homogenate using anti-HA antibody bound to magnetic beads. Total RNA was prepared from an aliquot of the same tissue homogenate. **B** Expression levels of Htt mRNA were slightly reduced in all cell types, significantly in cerebral vGluT2^+^ and PV^+^ neurons (* FDR ≤ 0.05; ** FDR ≤ 0.01; *** FDR ≤ 0.001). **C** HD-typical genes showed downregulation in cerebral cell types. **D** Differentially expressed genes (DEGs) (FDR ≤ 0.05) for each cell type. **E** Top five significantly enriched terms (FDR ≤ 0.05) among DEGs reveal cell type-specific responses to mHtt

To study cell type-specific translatomes we employed the RiboTag method [15]. It utilizes a knock-in mouse line in which the endogenous gene encoding large subunit ribosomal protein 22 (Rpl22) has been engineered such that activation by Cre recombinase results in a hemagglutinin (HA) antibody epitope being encoded on the C-terminus of Rpl22, and consequently HA-tagged ribosomes (RiboTag). From these samples, one can immunoprecipitate (IP) HA-tagged ribosomes from cell types of interest, and sequence the attached translating mRNAs, representing the translatome. To this end, we crossed Hdh^Q7/Q200^-Rpl22HA^flox/flox^ mice with homozygous Cre-driver lines to generate HD and control mice expressing RiboTag in targeted cell types. We targeted general populations of either GABAergic (Gad2^+^) or glutamatergic (vGluT2^+^) neurons in the cerebellum and the cerebrum, where the cerebrum is the remaining part after removal of the cerebellum and olfactory bulb from the brain. In the cerebrum, we also targeted the subset of GABAergic neurons expressing the neuropeptide parvalbumin (PV) (Fig. 3A). We have previously shown that these Cre lines direct RiboTag expression in the desired cell types [28]. Since the PV Cre line targets the same cerebellar cell types as the Gad2 line, and were thus redundant, the cerebellar PV samples were not processed.

Upon sequencing RiboTag-captured mRNAs, we found *Htt* mRNA levels were significantly reduced in cerebral PV^+^ and vGluT2^+^ neurons in HD mice, with the same trend in the other cell types (Fig. 3B), consistent with the histological analysis (Fig. 2F). Principal component analysis (PCA) of IP and total RNA samples showed a clear separation of RiboTag IP samples by targeted cell type, in contrast to total RNA samples (Fig. S4A, B), indicating that we successfully obtained cell type-specific translatomes from RiboTag IPs. This was confirmed by analyzing the expression of cell type-specific marker genes in comparison to total RNA samples (Fig. S4C, D). General GABAergic markers *Gad2*, *Slc32a1, Dlx2, and Lhx6* were enriched in cerebral Gad2^+^ and PV^+^, but not vGluT2^+^ neurons (Fig. S4C). PV^+^ samples showed enrichment for genes important for the development and function of cortical PV^+^ interneurons (*Syt2, Cplx1, Nek7, Ank1, Kcnc2, Gpr176, Pthlh*) [29–32], while marker genes for Gad2^+^/PV^−^ neurons, including genes with high striatal expression levels such as *Adora2a, Drd1, Drd2, Penk,* and *Ppp1r1b,* were depleted in PV^+^ samples, as were markers of other PV^−^ GABAergic interneuron subtypes (*Sst, Vip* and *Htr3a*). Cerebellar Gad2^+^ samples (Fig. S4D) showed enrichment of markers for Purkinje (*Pcp, Grid2, Esrrb, Foxp2*), Basket (*Kcna2*), Golgi (*Lgi2, Gdj2, Sorcs3*), and Stellate cells (*Grik3*), while cerebellar vGluT2^+^ samples showed enrichment for granule cell markers (*Gabra6, Cntn2, Zic1, Etv1, Nfia*) [33, 34]. As expected, astrocyte and microglia markers [35], were depleted in all IP samples compared to total RNA. Taken together, these results confirm the successful isolation of cell type-specific translatomes with RiboTag and the inclusion of expected neuronal populations in both targeted regions.

### Translatome analysis reveals cell type-specific responses to mHtt

Next, we performed differential gene expression analysis to study the cell type-specific responses of targeted neurons to mHtt. For cerebral neurons, we observed downregulation of known HD-associated neuronal genes, such *as Drd1, Drd2, Penk, Ppp1r1b, Pde10a, Arpp21*, and *Pcp4, all* commonly reported to be downregulated in both human HD patients and various HD mouse models [36–38] (Fig. 3C). Several of these genes show high striatal expression in early vulnerable populations i.e., Gad2^+^ SPNs, so the observed downregulation of these genes in cerebral Gad2^+^ neurons further supports a striatal phenotype in our HD mice at 9 months. Comparison of our cell type-specific data with bulk RNAseq of striata from the zQ175DN HD knock-in model [10] showed a highly significant overlap between gene expression changes detected in cerebral Gad2^+^ samples and bulk striatal tissue at all observed time points (Fig. S5A), further indicating that our S4-HdhQ200 mice have HD-typical phenotypes. There was also a significant overlap of bulk RNAseq data with our cell type-specific results for cerebellar Gad2^+^ and vGluT2^+^ neurons (Fig. S5B). Notably, these HD-associated genes were also downregulated in cerebral vGluT2^+^ or PV^+^ neurons, which may be explained in at least two ways. First, downregulation of striatal marker genes may occur not only in highly vulnerable striatal projection neurons but in other cell types as well, as shown in a recent mouse study for glutamatergic corticostriatal projection neurons, astroglia, and cholinergic interneurons [14]. Second, the downregulation of these genes may occur in regions beyond the striatum, which would be detected when multiple regions are combined.

**Fig. 4:**
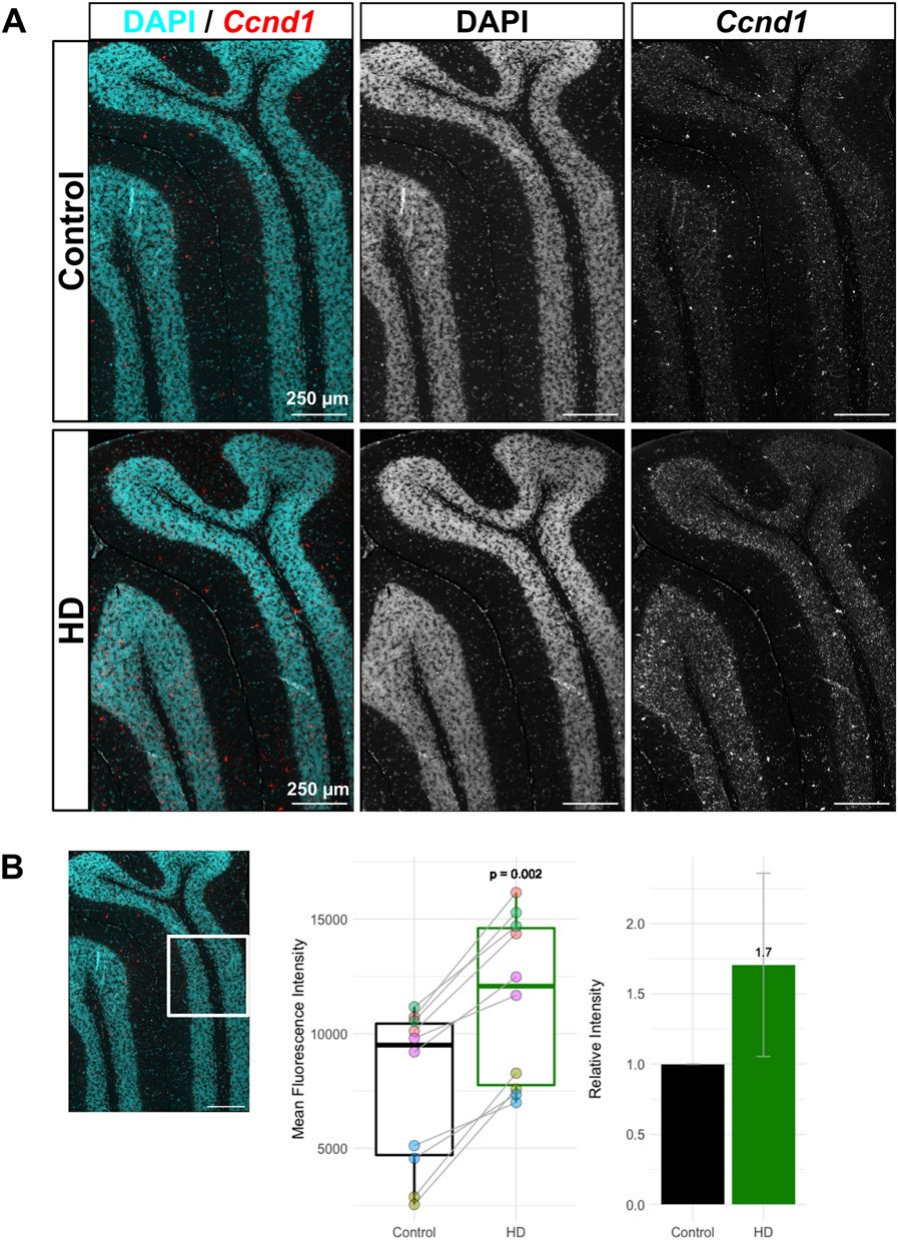
Ccnd1 ISH staining is increased in the granular layer of 9-month-old HD mice. **A** In situ hybridization for *Ccnd1* mRNA (red) shows increased signal in the anterior cerebellar lobules III-V in 9-month-old HD mice (bottom) compared to littermate controls (top). **B** Quantification of mean fluorescence intensity in the granule layer of lobule IV/V (white square). Analysis was performed on two measures obtained from five biological replicated per group. At 9 months, HD samples (n = 10, Shapiro-Wilks: W = 0.87, *p* = 0.11) show a significant 1.7-fold increase of *Ccnd1* staining compared to controls (n = 10, Shapiro-Wilks test: W = 0.83, *p* = 0.03), Wilcoxon rank sum test, *p* = 0.002, n = 10, matched control and HD samples are indicated by dot color and connecting line

Surprisingly, cerebellar vGluT2^+^ neurons showed the overall highest number of differentially expressed genes (DEGs) with 626 (Fig. 3D). To discover functional associations, we analyzed the overrepresentation of gene ontology (GO) terms in the biological process classes among cell type-specific DEGs, which revealed cell type-specific responses (Fig. 3E). DEGs of cerebral Gad2^+^ neurons were associated with dopamine transport (FDR = 0.009, rich factor = 0.07) and secretion (FDR = 0.02, rich factor = 0.07), ion transport (FDR = 0.009, rich factor = 0.1), downregulation of neuropeptides vasopressin and proenkephalin (*Vip, Penk*), and downregulation of several genes encoding neurotransmitter receptor components for dopamine (*Drd1*), glutamate (*Grm3*), acetylcholine (*Chrna6*), cannabinoid (*Cnr1*), and serotonin (*Htr4*) (Fig. S6A). Cerebral vGluT2^+^ neurons were enriched for gene sets associated with cognition, memory (FDR = 0.05, rich factor = 0.03), and locomotory behavior (FDR = 0.04, rich factor = 0.02), while PV^+^ neurons were associated with myelination (FDR = 0.004, rich factor = 0.13) and neurotransmitter uptake (FDR < 0.001, rich factor = 0.13). Top enriched pathways in cerebellar vGluT2^+^ neurons were related to exocytosis (FDR = 0.03, rich factor = 0.13) and vesicle fusion (FDR = 0.002, rich factor = 0.55; “regulation of vesicle fusion” FDR = 0.01, rich factor = 0.28) (Fig. 3E), driven by downregulation of genes encoding the central components involved in calcium-dependent synaptic vesicle fusion and exocytosis, such as complexin-encoding genes *Cplx2* and *Cplx3*, *Snap25,* and *Vamp2*, as well as differential expression of Ca^2+^ sensors *Syt7, Doc2b* and *Otof* (Fig. S6A). Cerebellar Gad2^+^ neurons, in contrast, indicated activation of mitotic cell cycle regulation, in particular G1/S transition (FDR = 0.004, rich factor = 0.017), with upregulation of cyclin D1 (*Ccnd1*), a regulator of G1/S progression, downregulation of antiproliferative p21/cyclin-dependent kinase inhibitor 1a (*Cdkn1a*) and polo-like kinase 5 (*Plk5*) (Fig. S6A). *Ccnd1* was also among the strongest upregulated genes in cerebellar vGluT2^+^ neurons (log2 fold change = 1.8, FDR < 0.001). This was validated by ISH for *Ccnd1* mRNA in tissue sections of 9 months old HdhQ200 mice (Fig. 4A), showing a 1.7-fold increase in *Ccnd1* staining in the granule layer in HD mice (Wilcoxon rank sum test, *p* = 0.002) (Fig. 4B). Therefore, the cerebellum shows a robust, cell type-specific response to the HD mutation as early as 9 months with a focus on pathways involving neurotransmission, vesicles, or cell cycle reentry.

### Cerebellar neurons show cell type-specific responses to mHtt

**Fig. 5:**
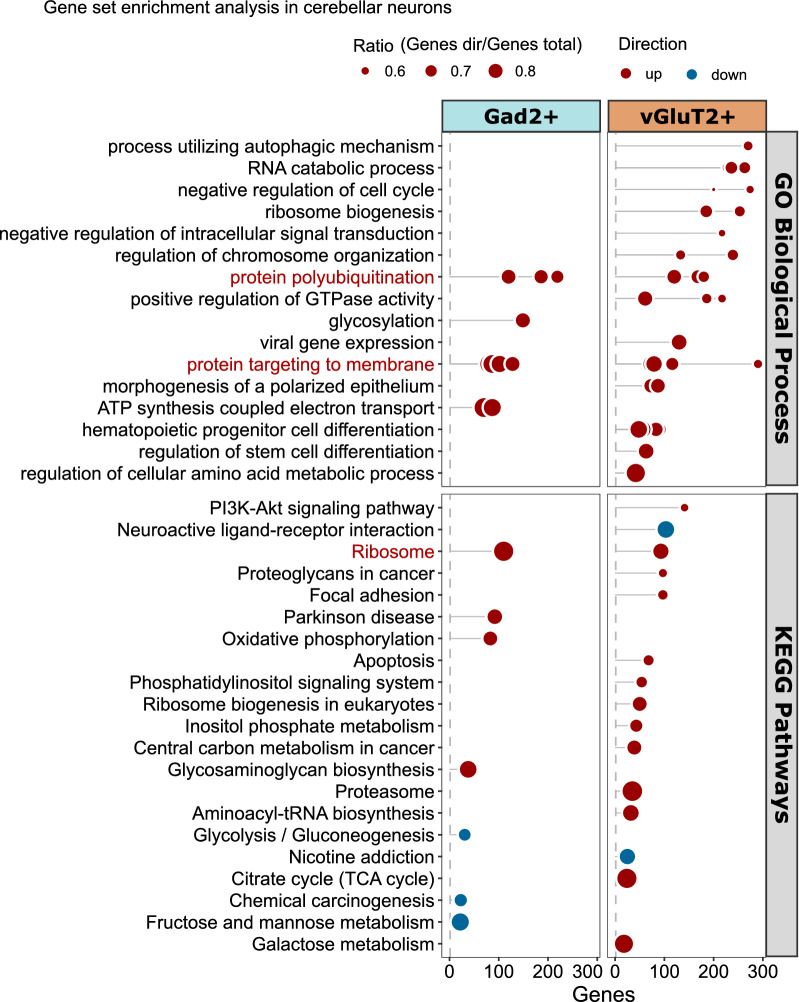
Cerebellar neurons show early and cell type-specific responses. Gene set enrichment analysis shows a largely cell type-specific response in Gad2^+^ and vGlut2^+^ cerebellar neurons, shared terms are indicated in red. GO terms were reduced to parent terms based on semantic similarity. The ratio indicates the relative number of enriched genes to gene set size

Given the high number of DEGs detected in cerebellar vGluT2^+^ neurons and that nearly all of the cerebellar Gad2^+^ DEGs were also changed in vGluT2^+^ cerebella, we conducted additional analyses of expression changes. Comparison of DEGs between cerebellar cell types revealed that the shared DEGs changed with the same directionality (Fig. S6B), again suggesting a similar response. We next performed gene set enrichment analysis (GSEA) on both cell types, using six different statistical methods to calculate enrichment using a consensus score (CS) to rank results [21]. GSEA is a useful tool to detect pathways that have a coordinated response even when individual genes of that pathway have small and statistically insignificant changes. Interestingly, the GSEA indicated that, despite sharing the majority of the DEGs with cerebellar vGluT2^+^ neurons, cerebellar Gad2^+^ neurons had a remarkably different response (Fig. 5). Genes associated with “translation and co-translational targeting of proteins to membrane” and endoplasmic reticulum (CS = 1) were enriched in both cerebellar cell types (Fig. S7). However, Gad2^+^ neurons uniquely showed upregulation of protein glycosylation-related gene sets (CS = 1.5) and ATP synthesis (CS = 1), oxidative phosphorylation (CS = 5.5), and Parkinson’s disease pathway (CS = 1), suggesting an increase of mitochondrial function in these neurons. In contrast, vGluT2^+^ cerebellar neurons made a cell type-specific response in the form of upregulation of cell differentiation-associated gene sets (CS = 1), cell cycle regulation (CS = 1) and chromosome organization (CS = 1), autophagy (CS = 1), metabolic processes, upregulation of PI3K-Akt signaling pathway genes (CS = 12) and apoptosis (CS = 12). This suggests that, despite superficial similarities, cerebellar cell types activate different pathways in response to mHtt. Additionally, the enrichment of genes associated with differentiation, PI3K-Akt signaling, and apoptosis, together with the upregulation of *Ccnd1*, may indicate that cell cycle regulation is affected in cerebellar vGluT2^+^ neurons. Interestingly, we found significant overlaps of DEGs identified in both vGluT2^+^ and Gad2^+^ cerebellar neurons with DEGs in the cerebellum of a knock-in mouse model of Spinocerebellar ataxia 1 (SCA1) [OMIM:164400], an autosomal dominant disease caused by expansion of a polyglutamine encoding CAG repeat in the Ataxin1 gene (*Atxn1*) (Fig. S8) [39]. This revealed that genes detected in both vGluT2^+^ and Gad2^+^ cerebellar cell types were also among the earliest DEGs in SCA1 (Fig. S8).

### Polycomb repressor complex 1 (PRC1) protein genes are upregulated in cerebellar vGluT2^+^ neurons

**Fig. 6:**
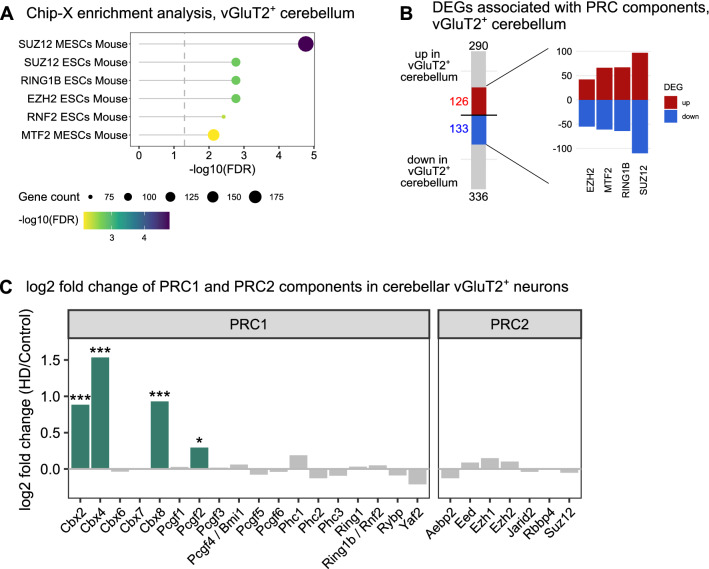
Polycomb repressor complex 1 (PRC1) proteins are upregulated in cerebellar vGluT2^+^ neurons. **A** Overrepresentation analysis against ChEA ChIPseq gene sets from mouse embryonic stem cells (ESCs/MESCs) show DEGs detected in vGluT2^+^ neurons are overrepresented among targets associated with polycomb repressor complex (PRC) proteins (FDR ≤ 0.01). **B** PRC-associated DEGs show similar distribution between up and down-regulated DEGs. **C** PRC1 protein genes *Cbx2, Cbx4, Cbx8* and *Pcgf2* are significantly upregulated in cerebellar vGluT2^+^ neurons (green) in HD, whereas PRC2 core protein genes (*Eed, Ezh1, Ezh2, Suz12*) and associated factors (*Aebp2, Jarid2, Rbbp4*) are not differentially expressed. (* FDR ≤ 0.05; ** FDR ≤ 0.01; *** FDR ≤ 0.001)

We next investigated if the differential expression patterns we observed in cerebellar vGluT2^+^ neurons could be driven by specific transcription factors using the ChIP Enrichment Analysis (ChEA) database [22] to perform overrepresentation analysis against the transcription factor targets determined by Chip-X. This analysis revealed that 259 out of 626 DEGs were among target genes of polycomb repressor complex 2 (PRC2) components SUZ12 or EZH2, PRC1 core component RING2B/RNF2, or PRC-associated factor MTF2 (Fig. 6A). Of these 259 genes, 218 were associated with PRC2 core proteins EZH2 (97 DEGs) and SUZ12 (207 DEGs), and 131 DEGs with PRC1 protein RING2B/RNF2 showing near equal distribution of up- and downregulated DEGs (Fig. 6B). Levels of PRC2 core components and associated factors were not affected, but we found increased expression of canonical PRC1 complex components chromobox proteins *Cbx2, Cbx4, Cbx8* and polycomb group ring finger 2 (*Pcgf2)* in cerebellar vGluT2^+^ neurons (Fig. 6C). Although PRC2 regulation is impaired in HD [40, 41], little is known on the involvement of PRC1 in HD. However, given the proposed roles of CBX proteins in regulating cell cycle progression across various checkpoints [42], these findings further support the view of aberrant cell cycle regulation in cerebellar vGluT2^+^ neurons.

## Discussion

Here we characterized an HdhQ200 knock-in mouse model in a new background, 129S4. Behavioral and neuropathological characterization of this mouse model showed widespread Htt^+^ NIIs and downregulation of *Htt* and *Ppp1r1b* mRNAs by 9 months, with a very mild behavioral phenotype at later disease stages. These histological changes motivated us to use RiboTag to further analyze 9 months old HD mice, and to identify, for the first time, cell type-specific translatome responses in the cerebellum at an early disease stage.

Surprisingly, our translatome analysis revealed widespread changes in cerebellar vGluT2^+^ neurons, a population consisting predominantly of granule neurons. This result was unexpected, as granule cells are typically considered resistant in HD. Instead, compromised function and loss of Purkinje cells is the cerebellar phenotype most described in HD patients and mouse models [2, 3, 5]. Functional analysis of DEGs in cerebellar vGluT2^+^ neurons revealed vesicular fusion and exocytosis as predominantly enriched biological processes, suggesting neurotransmitter release and synaptic signaling may be affected. GSEA further suggested the upregulation of genes related to differentiation, cell cycle regulation, and energy metabolism which are largely congruent with previous data on gene expression signatures in the cerebellum. Those studies also reported chromatin-binding, mitochondrion, synapse and proteasome-related genes [13], although in some cases the directionality of change differed from results in our analysis. This discrepancy may be due to differences in disease severity or methodology. Further, translating the directionality of gene expression changes to mechanistic changes is complex as it does not consider the function of gene products within a given pathway (i.e., inhibitory or activating).

One of the most notable gene expression changes was the strong upregulation of *Ccnd1* in both cerebellar vGluT2^+^ and Gad2^+^ neurons, which was confirmed by ISH staining. Ccnd1 is a core regulator of G1/S transition, and its upregulation is associated with cell cycle reentry (CCR). CCR of postmitotic neurons has been proposed as an early event in progressive neurodegeneration which can be triggered by dysregulation of various mechanisms, several of which are known to be affected in HD, e.g., altered levels of neurotrophic factors, oxidative stress, unrepaired DNA damage, or dysfunction of the ubiquitin–proteasome system [43]. Activation of Notch signaling and the downstream Akt/GSK3b pathway in response to an excitotoxic stimulus can cause CCR in mature neurons by Ccnd1 upregulation driving G1/S transition, which can in turn prompt neurons to enter apoptosis [44]. Indeed, Ccnd1 upregulation has also been observed in striatal neurons in response to 3-Nitropropionic acid treatment [45, 46], a model replicating HD-like striatal neuron loss. This explanation is in line with our observation of upregulation of PI3K-Akt signaling and apoptosis-related genes in cerebellar vGluT2^+^ neurons, suggesting CCR may also occur in cerebellar vGluT2^+^ neurons, at early, pre-symptomatic disease stages.

Another important finding was the high percentage of DEGs in cerebellar vGluT2^+^ neurons associated with PRC components. HTT is a known facilitator of PRC2 [40, 47, 48] and neurons expressing mHtt show aberrant chromatin landscapes already at early developmental stages, which may result in reduced neuronal fitness [47], and PRC2 silencing is important for maintaining cell identity in differentiated neurons [49, 50]. Given the established role of PRC2 in HD, the high number of PRC2-associated DEGs in cerebellar vGluT2^+^ neurons suggests an epigenetic mechanism underlies the observed changes. In contrast, little is known on whether PRC1 plays a role in HD. Therefore, we were surprised to see upregulation of several canonical PRC1-related genes in cerebellar vGluT2^+^ neurons. Cbx family proteins (Cbx2, 4, 6, 7, 8) form core elements of the canonical PRC1 complex recognizing H3K9me2 marks. Beyond this, they control cell cycle, proliferation, and senescence with Cbx2, Cbx4 and Cbx8 having distinct roles in regulating cell cycle progression [42]. Cbx2 and Cbx8 may further act as positive regulators of axonal regeneration [51], while Cbx4, a SUMO E3-ligase, is essential for SUMOylation of Bmi1/Pcgf4, facilitating its recruitment and accumulation at DNA damage sites [52]. Additionally, Cbx2 and Cbx8 proteins also interact with REST, a master regulator of neuronal gene expression and interaction partner of Htt, affecting PRC1 binding patterns and REST-mediated transcriptional repression [53]. This functional diversity of CBX proteins and their involvement in central mechanisms that are disturbed in HD, such as cell cycle regulation and DNA damage repair, provides several interesting mechanisms by which upregulation of these genes may be important in the response of granule cells, opening new avenues for future research.

Interestingly, our results from specific cerebellar neurons of HD mice also had considerable overlap with bulk RNAseq data from cerebella of a SCA1 mouse model [39]. Most notably, the pronounced downregulation of *Igfbp5* in the cerebellum was also described as a robust feature in SCA1 and SCA7 knock-in mice [54]. Igfbp5 downregulation increases IGF1 availability, which may trigger pro-survival pathways via IGF1R signaling to support neuronal function [54, 55]. This suggests mechanistic similarity between the polyglutamine diseases HD and SCA1, a finding that would be interesting in light of an increased understanding of cerebellar involvement in HD pathology and symptoms in various HD patients.

Further research is required to determine the mechanism and consequences of the translatome changes observed in our experiments, especially regarding the question whether the observed responses are detrimental or beneficial to neurons. However, the pronounced cell type-specific response in cerebellar vGluT2^+^ neurons early in disease offers new and interesting insights into early disease response in the cerebellum. While the presence and extent of cerebellar dysfunction in HD appears to be more variable than striatal perturbations, it also opens the possibility of finding new potential therapeutic targets and interventional strategies. Indication of cerebellar compensation in early stages of HD make this approach particularly appealing.

Finally, one of the goals of the study was to reveal new insight into the phenomenon of selective vulnerability. While we uncovered molecular pathways underpinning the cerebellum’s response to HD, the understanding of the broader topic of selective vulnerability was only modestly enhanced. Nonetheless, we found it interesting that the thalamus is the brain region with the second highest Htt expression but is a region with few Htt aggregates, while the striatum and cerebellum are low expressors but are highly prone to form aggregates, especially NIIs. These observations suggest that HD-vulnerable brain regions are poorly equipped to handle the Htt protein in general, and the inclusion of a long polyglutamine stretch makes that struggle more severe.

### Limitations

One limitation of our experimental setup is that we studied ribosome-bound, translating mRNA and thus are not able to conclude whether observed changes occur due to changes at a transcriptional or translational level. This could be addressed by methods such as the Tagger mouse line [56], enabling cell type-specific assessment of four levels of gene expression. Another limitation is that we studied cell populations with various degrees of heterogeneity. This may partially explain the high number of DEGs in cerebellar vGluT2^+^ neurons, arguably the least heterogeneous cell type analyzed in this study. However, in a previous study using the same experimental approach, we did not observe strong changes in the cerebellar vGluT2^+^ neurons [20], indicating this result is not an artifact but reflects the biological response of this cell type in HD. A third limitation is that the cerebrum is a mix of many regions, and changes in specific regions may have been obscured. Importantly, this study ran parallel with another study of two genetic prion diseases [20]. The cerebellum is affected in both prion models, while the thalamus is also targeted in one model and the hippocampus in the other. Since the cerebellum was an important focus in both studies, we chose to conserve resources by not sub-dissecting the cerebrum into multiple regions. A final limitation is the use of a genetic background not widely used in HD research. In previous experiments, we observed that a subset of mice with a C57BL/6J background was hyperactive at night, which we thought could impact gene expression [19]. To this end, the HdhQ200 model was backcrossed to a 129S4 background. This model has a very mild behavioral phenotype, like other knock-in HD mouse models. Importantly, histological assessment and translatome analysis revealed an HD-typical phenotype and many gene expression changes we detected have been detected in other studies of HD mice. Although direct comparison of our results to those of other studies require consideration of possible genetic background effects, the existence of the HdhQ200 model on a new genetic background may be useful for identifying genetic modifiers.

## Supplementary Information

**Additional file 1:** **Figure S1**. Overview of HD and Control mice used in experiments. **Figure S2**. Distribution of Htt+ aggregates and Htt expression in HD mouse brains. **Figure S3**. Reactive gliosis is not prominent in HD mouse brains. **Figure S4**. RiboTag Immunoprecipitation yields cell type-specific translatome samples. **Figure S5**. RiboTag IP samples show significant overlap with bulk RNA data. **Figure S6**. Overrepresentation analysis reveals cell type-specific responses in targeted neuronal subtypes. **Figure S7**. GSEA reveals cell type-specific response of cerebellar neurons. **Figure S8**. DEGs of cerebellar neurons show significant overlap with genes detected in SCA1 cerebellum. **Supplementary Material and Methods**.

## Data Availability

All raw and processed sequencing data generated in this study have been submitted to the NCBI Gene Expression Omnibus (GEO; https://www.ncbi.nlm.nih.gov/geo/) under accession number GSE199837.

## Abbreviations

CCR: Cell cycle re-entry
DEG: Differentially expressed gene
FDR: False discovery rate
GO: Gene ontology
GSEA: Gene set enrichment analysis
HA: Hemagglutinin
HD: Huntington’s disease
HTT: Huntingtin
IGF: Insulin-like growth factor
IP: Immunoprecipitation
ISH: In-situ hybridization
mHTT: Mutant Huntingtin
NHS: Normal horse serum
NII: Neuronal intranuclear inclusion
ORA: Overrepresentation analysis
PRC: Polycomb repressive complex
PV: Parvalbumin
rpm: Revolutions per minute
RT: Room temperature
SCA: Spinocerebellar ataxia
SD: Standard deviation
SPN: Spiny projection neuron
