# Supplementary material for: Cerebellar granule neurons induce Cyclin D1 before the onset of motor symptoms in Huntington’s disease mice
Source: Acta Neuropathol Commun. 2023 Jan 20;11:17. doi: 10.1186/s40478-022-01500-x (PMC9854201; doi:10.1186/s40478-022-01500-x)
Supplement: Supplementary file 1 — Additional file 1: Figure S1. Overview of HD and Control mice used in experiments. Figure S2. Distribution of Htt+ aggregates and Htt expression in HD mouse brains. Figure S3. Reactive gliosis is not prominent in HD mouse brains. Figure S4. RiboTag Immunoprecipitation yields cell type-specific translatome samples. Figure S5. RiboTag IP samples show significant overlap with bulk RNA data. Figure S6. Overrepresentation analysis reveals cell type-specific responses in targeted neuronal subtypes. Figure S7. GSEA reveals cell type-specific response of cerebellar neurons. Figure S8. DEGs of cerebellar neurons show significant overlap with genes detected in SCA1 cerebellum. Supplementary Material and Methods. [file 40478_2022_1500_MOESM1_ESM.docx]

**Supplemental material**

**Cerebellar granule neurons induce Cyclin D1 in** **an early stage of Huntington’s disease**

**Authors**

Susanne Bauer^1^, Chwen-Yu Chen^1^, Maria Jonson^1^, Lech Kaczmarczyk^1,2^, Srivathsa Magadi^1^, Walker S. Jackson^1,2,#^

**Affiliations**

1. Wallenberg Center for Molecular Medicine, Department of Biomedical and Clinical Sciences, Linköping University, Linköping, Sweden
2. German Center for Neurodegenerative Diseases, Bonn, Germany

# corresponding author’s contact information: Linköping University Hospital, room 463.10.30, Linköping, Sweden, Tele: +46 13 28 66 21, email: walker.jackson@liu.se

**Running title:**

**Cerebellar response in early Huntington’s disease**

**Table of contents**

Pg. 3 Supplementary figures

Pg. 3 Figure S1: Overview of HD and Control mice used in experiments.

Pg. 4 Figure S2: Distribution of Htt^+^ aggregates and Htt expression in HD mouse brains

Pg. 6 Figure S3: Reactive gliosis is not prominent in HD mouse brains

Pg. 8 Figure S4: RiboTag Immunoprecipitation yields cell type-specific translatome samples.

Pg. 10 Figure S5: RiboTag IP samples show significant overlap with bulk RNA data.

Pg. 11 Figure S6: Overrepresentation analysis reveals cell type-specific responses in targeted
neuronal subtypes.

Pg. 12 Figure S7: GSEA reveals cell type-specific response of cerebellar neurons.

Pg. 13 Figure S8: DEGs of cerebellar neurons show significant overlap with genes detected in SCA1 cerebellum.

Pg. 14 Supplementary Material and Methods

Pg. 14 Mouse models

Pg. 15 Behavior

Pg. 17 Neuropathology

Pg. 20 RiboTag RNAseq

Pg. 21 Bioinformatic analysis

Pg. 23 References

**Supplemental Figure 1**

**Figure S1: Overview of HD and Control mice used in experiments.** Number of female (F) and male (M) HD and Control mice used in different experiment. Histological characetization was performed on different cohorts of littermate HD and Control mice aged 4, 9 and 18 months. Behavioral characterization was performed on the same cohort of age-matched HD and littermate Controls between the ages of 3 to 18 months. RNA for RiboTag experiments was obtained at 9 months. We analyzed three cohorts differentiated by the targeted neuronal subtype (PV, Gad2, vGluT2). For mice expressing Rpl22-HA in Gad2 and vGluT2 cells, cerebrum and cerebellum were analyzed. Total RNA was sequenced from a subset of mice from which RiboTag RNA was isolated.

**Supplemental Figure 2**


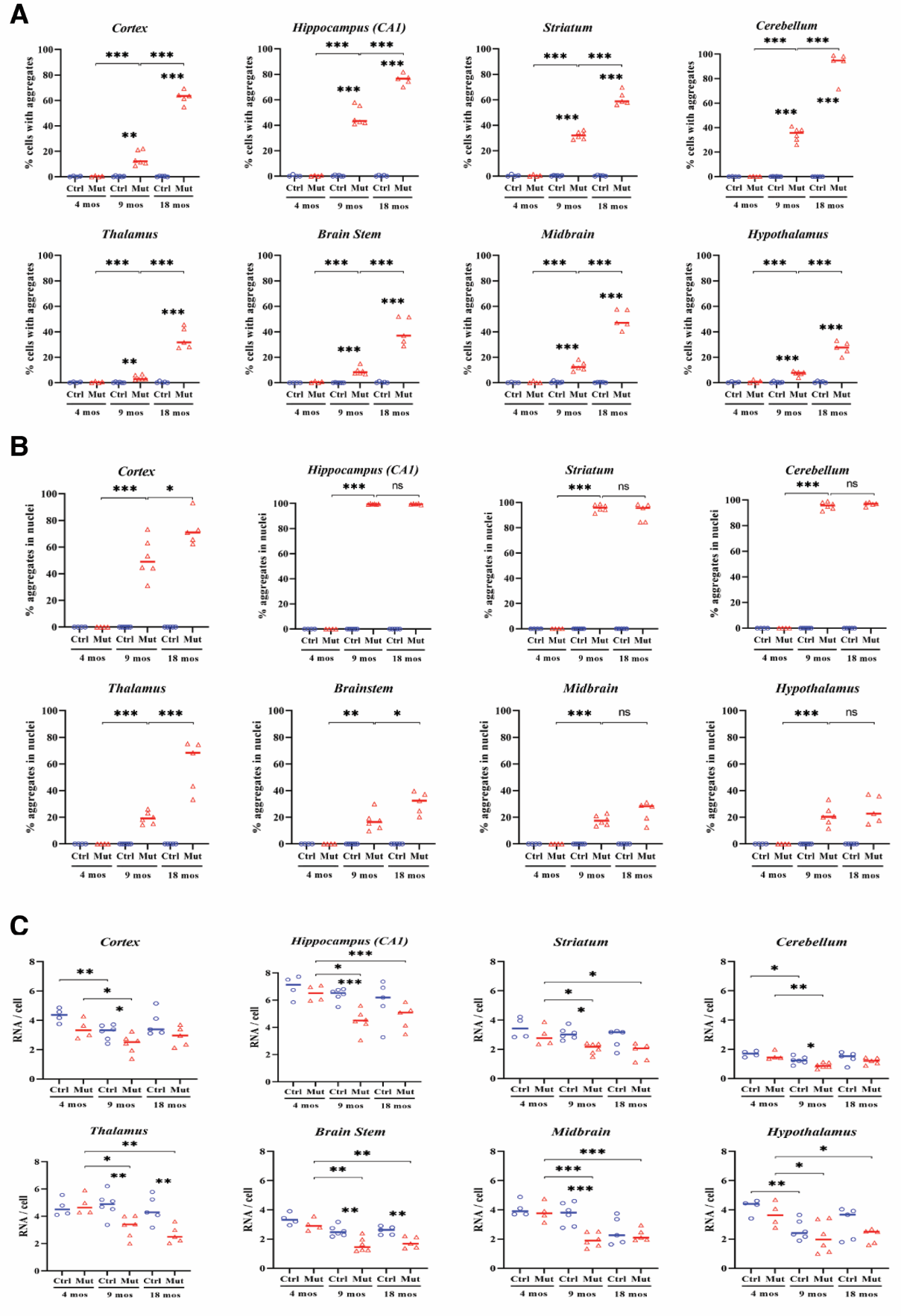


**Figure S2: Distribution of Htt^+^ aggregates and Htt expression in HD mouse brains**

These charts have the same data as Fig 2 but here the data are less condensed, each chart represents a single region, and the results of statistical analyses are presented. Data were analyzed by unpaired t-tests, and comparisons with p < 0.05, p < 0.01, and p < 0.001 are represented by *, **, and ***, respectively. Corrections for multiple testing were not made. **A)** The percent of cells with Htt aggregates, was calculated by dividing the number of cells by the number of aggregates, resulting in > 100% for cerebellum. **B)** The percent of aggregates that are within DAPI staining, and thus nuclear. **C)** The number of detected Htt RNA molecules per cell.

**Supplemental Figure S3**

**Figure S3: Reactive gliosis is not prominent in HD mouse brains**

**A)** GFAP labels many immature astrocytes, some mature astrocytes, and most astrocytes undergoing reactive gliosis. Here staining is similar in HD mice at all ages. In contrast, in the brain of an APPPS1 mouse modeling Alzheimer’s disease (AD), reactive gliosis is obvious.

**B)** Iba1 labels resting and activated microglia. In activated microglia the processes shorten and thicken resulting in a more intense staining. Sections stained with Iba1 were parallel with those labeled with GFAP antibodies in A (i.e., from the same brains, presenting the same regions). Iba1 produces a similar labeling for all ages of HD brains but a strong labeling for the AD brain. Control brains also showed no gliosis. Regions labeled “hippocampus” also include cortex, plus other areas, and regions labeled “striatum” also include thalamus, plus other areas.

**Supplemental Figure 4**


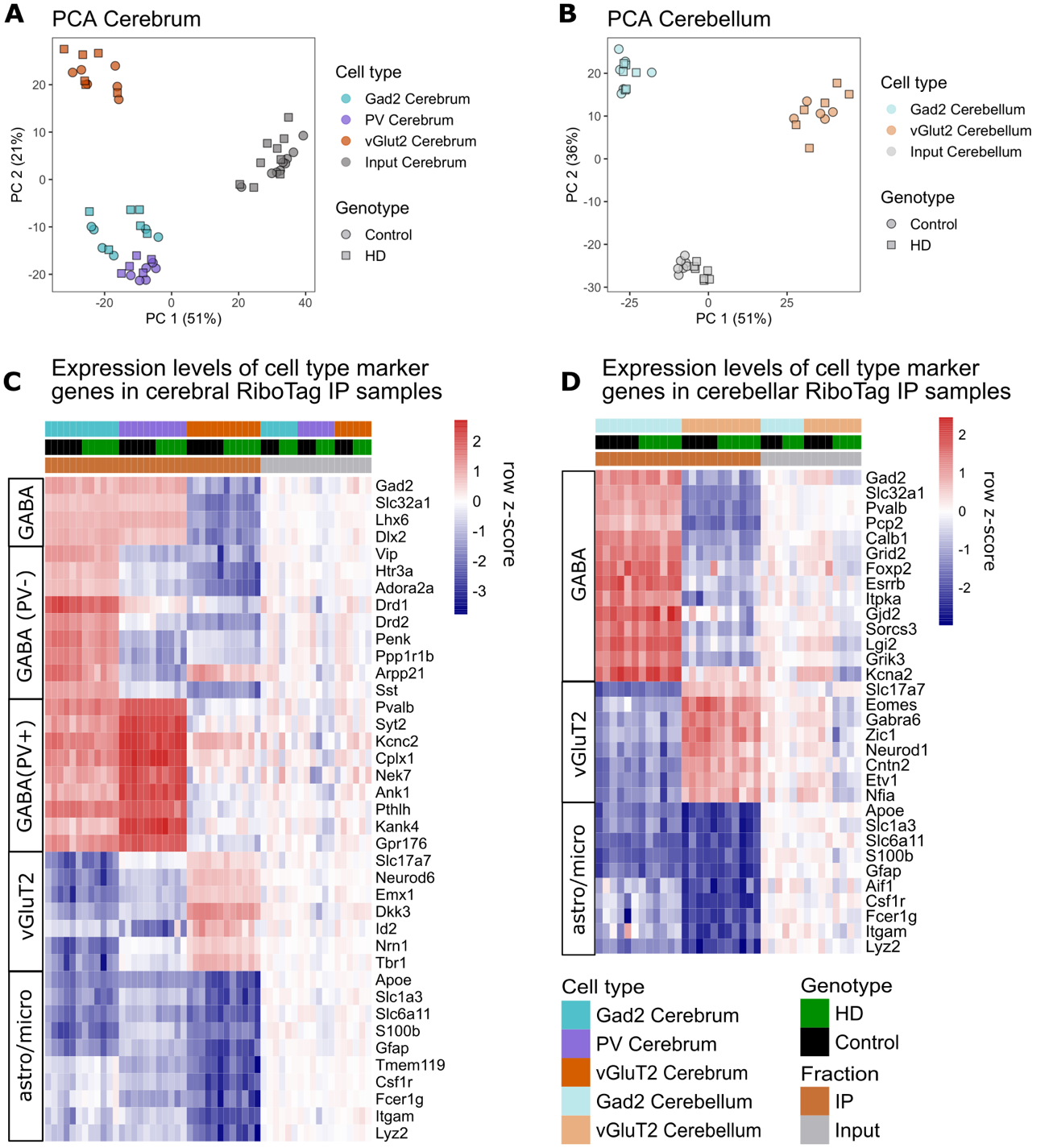


**Figure S4: RiboTag Immunoprecipitation yields cell type-specific translatome samples. (A, B)** Principal component analysis of top 500 expressed genes shows samples obtained by RiboTag IP from Cerebrum (A) or Cerebellum (B) cluster by cell type, whereas samples obtained from bulk tissue without applying RiboTag show no separation by cell type. **(C, D)** RiboTag samples obtained from cerebral (C) and cerebellar (D) tissue samples show enrichment of marker genes specific for targeted cell types when compared to total RNA obtained from the same tissue region prior to performing RiboTag IP. This confirms enrichment of cell type-specific mRNA through RiboTag.

**Supplemental Figure S5**

**
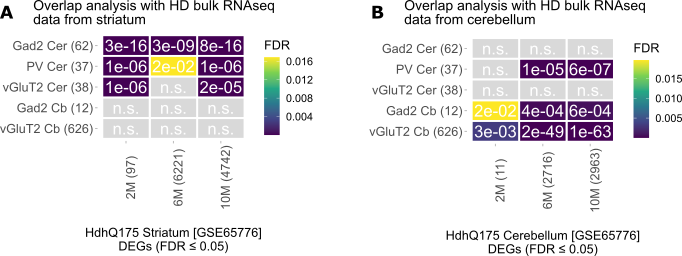
**

**Figure S5: RiboTag IP samples show significant overlap with bulk RNA data.** (A) DEGs identified in the three cerebral cell types, but not in cerebellar cell types, show significant overlap with striatal bulk RNA tissue. (B) Cerebellar Gad2^+^ and vGluT2^+^ neurons show significant overlap in DEGs with cerebellar bulk RNAseq data even at the earliest time point.

**Supplemental Figure S6**


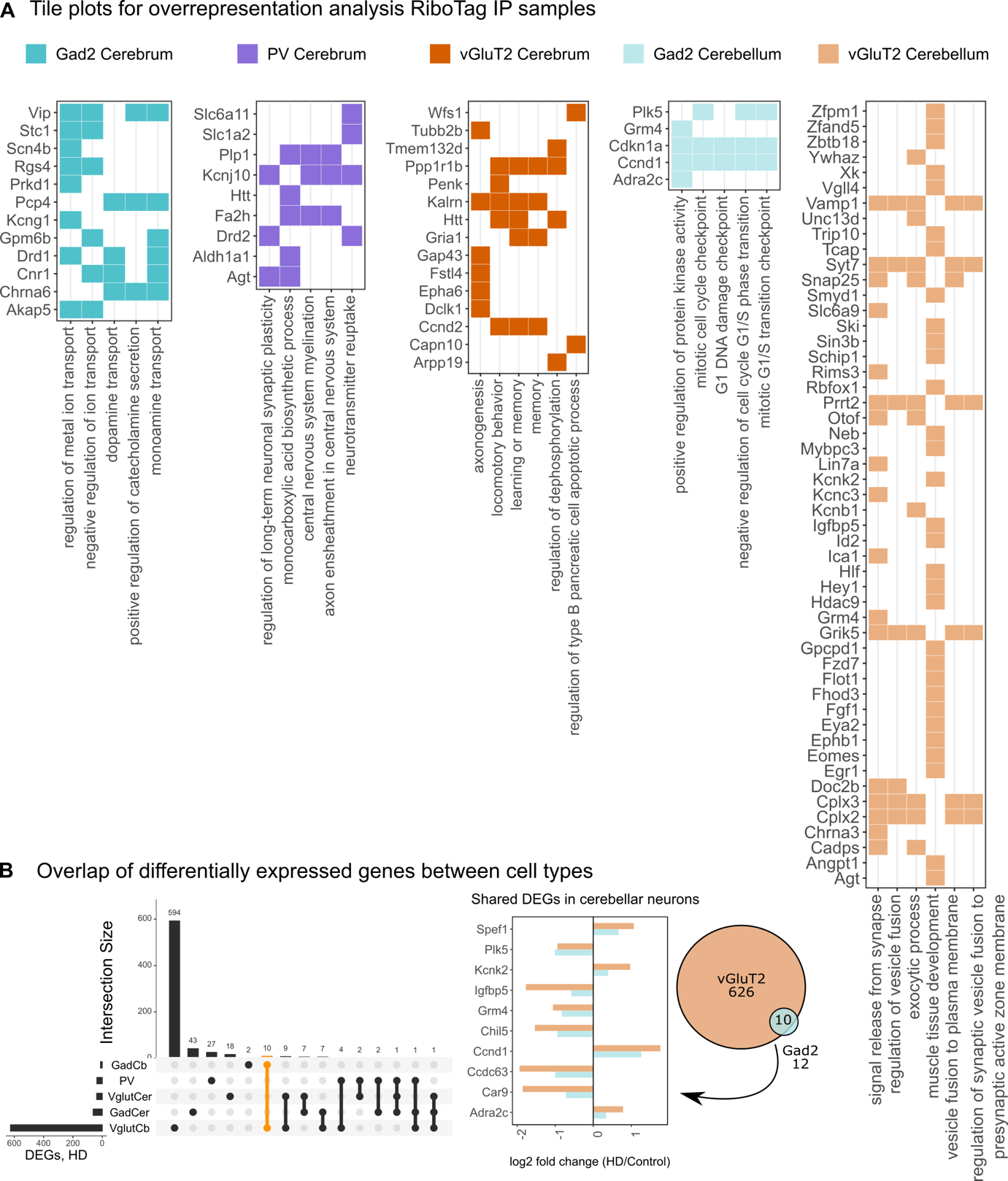


**Figure S6: Overrepresentation analysis reveals cell type-specific responses in targeted neuronal subtypes. (A)** Differentially expressed genes (DEGs) associated with the top 5 overrepresented gene sets (FDR ≤ 0.05) for each cell type. **(B)** 10 of 12 DEGs in cerebellar Gad2^+^ neurons are shared with cerebellar vGluT2^+^ neurons showing same directionality of change.

**Supplemental Figure S7**


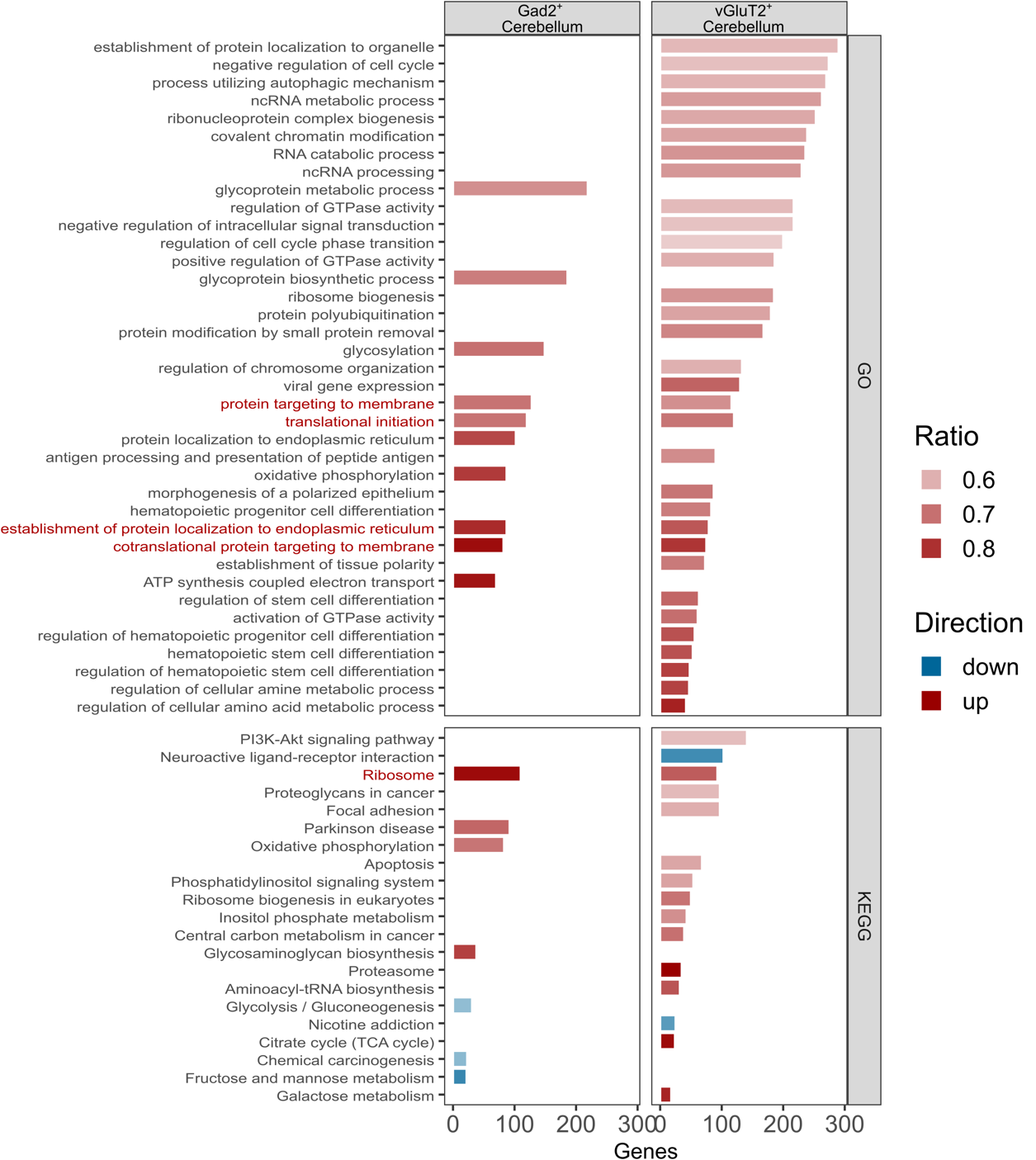


**Figure S7: GSEA reveals cell type-specific response of cerebellar neurons.** Analysis was performed using six different statistical methods to calculate directional enrichment for GO Biological Process terms and mouse KEGG pathways in GABAergic (Gad2^+^) and glutamatergic (vGluT2^+^) neurons of the cerebellum in HdhQ200 mice at 9 months. Gene sets which were found significantly (FDR ≤ 0.05) enriched by at least two methods were included. Terms shared between cell types are indicated in red. Ratio indicates the relative number of enriched genes to gene set size.

**Supplemental Figure S8**

**
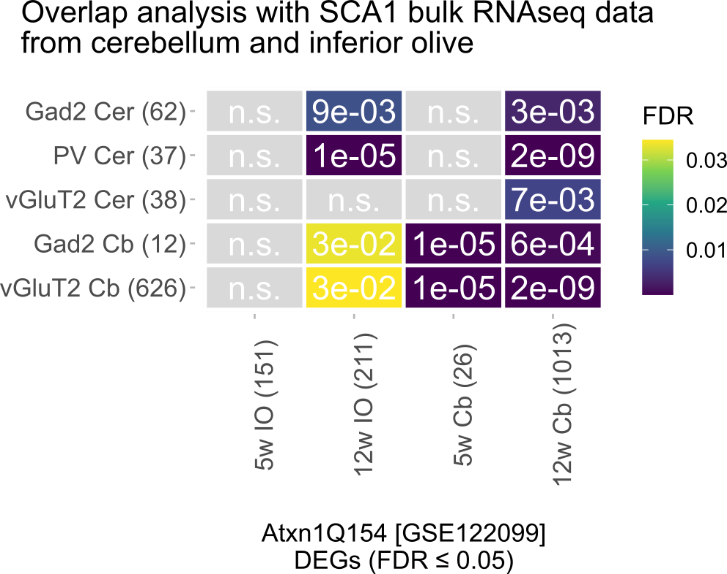
**

**Figure S8: DEGs of cerebellar neurons show significant overlap with genes detected in SCA1 cerebellum.** DEGs detected in both Gad2^+^ and vGluT2^+^ neurons showed significant overlap with DEGs from bulk cerebellar tissue (Cb) of a mouse model of spinocerebellar ataxia 1 (SCA1) in early disease (5 weeks), but not with DEGs obtained from inferior olive (IO). FDR-adjusted p-values are shown.

**Supplementary Material and Methods**

**Mouse models**

**Animal breeding.**

All mice were congenic on a 129S4 background. The HdhQ200 mouse line was derived from the HdhQ150 mouse line originally developed using an embryonic stem cell line derived from a 129P2 mouse [1]. During the development of the allele, the CAA codon naturally present in the murine gene was removed and a long CAG repeat inserted such that the CAG repeat is uninterrupted and no human *HTT* sequence is present. The HdhQ150 mouse line was subsequently backcrossed to C57Bl/6J and the repeat length was expanded to approximately 200 CAGs through selective breeding [2]. We imported these HdhQ200 mice to our colony in Germany and began backcrossing to 129S4. Single nucleotide polymorphism (SNP) profiling was carried out periodically to select breeders with the most 129S4 genomic content [3]. Hdh^Q7/Q200^ mice were assigned to the “HD” groups and Hdh^Q7/Q7^ littermates were assigned to the “control” groups.

For experimental HD mice, a PCR of genomic DNA was performed using FAM-labeled genotyping primers. Sizing of CAG repeat length was done on a 3500 Genetic Analyzer (Applied Biosystems, Waltham, MA) in reference to a GeneScan LIZ1200 sizing standard (Applied Biosystems).

Genotyping primer Hdh: fw CCCATTCATTGCCTTGCTG; rev: GCGGCTGAGGGGGTTGA

**For RiboTag experiments**, HdhQ200 mice at generation 7 (>99% 129S4) were crossed three times to homozygous RiboTag mice (B6N.129-Rpl22^tm1.1Psam^/J, line #011029; Jackson Laboratory, Bar Harbor, ME), that were previously [4] bred and used on the 129S4 background (> 99.5% 129S4) to establish Hdh^Q200/Q7^/Rpl22-HA^flox/flox^ mice. Cre-driver lines included vGluT2-IRES-Cre [5] (Slc17a6^tm2(cre)Lowl^/J, line #016963; Jackson Laboratory), Gad2-IRES-Cre [6] (Gad2^tm2(cre)Zjh^/J, line #010802; Jackson Laboratory), and PV-IRES-Cre [7] (B6;129P2-Pvalb^tm1(cre)Arbr^/J, line #008069; Jackson Laboratory), each backcrossed to be > 99% 129S4 [3]. To obtain experimental animals, male Hdh^Q7/Q200^/Rpl22-HA^flox/flox^ mice were crossed with female Hdh^Q7/Q7^ mice homozygous for Cre, resulting in all offspring being heterozygous for both Cre and RiboTag. HD and control mice were sacrificed at approximately 9 months of age (average age: 41.5 weeks, SD: 1.8; range: 39-45 weeks) by carbon dioxide asphyxiation. Mouse brains were separated into hemispheres along the midline. One hemisphere was preserved for formalin-fixed paraffin embedding for immunohistochemistry or RNA in situ hybridization as described below. The olfactory bulb was removed from the second hemisphere and cerebellum and cerebrum were separated by dissection, flash frozen on dry ice, and stored at -80 to -72 °C.

**For behavioral experiments**, HdhQ200 mice were bred to 129S4 to generation 12 at which point a SNP analysis found 347 of 347 informative SNPs were derived from 129S4 [3]. This strain was subsequently bred to 129S4 mice (internally called 129BD) carrying the Disrupted in schizophrenia 1 (*Disc1*) and non-agouti genes from C57Bl/6NTac mice that were 99.8% 129S4. This *Disc1* gene was brought in to eliminate the possibility of the small deletion in the 129S4 derived gene could impact neurodegeneration, even though an in-depth sleep and electroencephalography study indicates the genes are equivalent [8]. The non-agouti gene was brought in to enhance light contrast between mice and bedding material for future video-based behavioral studies [9].

**Behavior**

Seven 129S4 control mice (3 male, 4 female) and 12 Hdh^Q7/Q200^ mice (7 male, 5 female) were tested between the ages of three to 18 months. Animals were weighed monthly, and motor tests were performed every two to three months. All experiments were conducted during the light phase.

**Rotarod:** The accelerating rotarod (ENV-574M, Mead Associates Inc, Fairfax, VT) was used to assess motor coordination and balance. The rotarod was divided into five separate lanes by concentric discs, preventing visual or physical contact. Five mice were tested at a time and the latency to fall was recorded.  **Training:** Prior to the first test at three months of age, the animals were trained for two consecutive days. Each training session lasted for 300 seconds. If the mouse fell during the training session it was put back onto the rod until it had fallen three times or stayed on for at least 60 seconds. **Testing:** Animals were tested once every two to three months on the accelerating rotarod for 300 seconds and the latency to fall was recorded. If a mouse fell within the first 30 seconds of the experiment, it was returned to the rod and the experiment was continued. The speed of the rotation accelerated every 30 s from 4 revolutions per minute (rpm) to 40 rpm. If a mouse remained on the rod for the whole test period, it was removed from the rod and given a score of 300 seconds.

**Balance beam:** The balance beam was used to determine abnormalities in coordinated movements and balance. One mouse at a time was tested. In this test, the mouse was required to cross a raised narrow beam to reach a safe location (a black box) at the high end. The beam was 1 m in length, 17° angle of ascent and with a 1.5 cm to 0.5 cm taper across the width. The increased slope and the black safety box at the goal promote spontaneous moving uphill and reduces freezing on the beam. **Training:** Prior to the first test at three months of age animals were trained for two consecutive days. On the first day of training, the mouse was placed on the beam initially close to the safe box and then with increased distance to the box until the animal was at the start of the beam. The second day of training consisted of two trials. For both trials the mouse was placed at the start of the beam and had to traverse the beam to reach the box at the top end. **Testing:** Testing was conducted by two consecutive trials for each time point. The animal was placed at the start of the beam and the time it took to traverse the beam to reach the box at the top end was recorded. Between the two trials mice were put into a burrow arena (details below) for one hour.

**Burrowing:** The burrow was made of a 20 cm long black plastic tube with a diameter of 7 cm. One end of the tube was closed with a plug, while the other end was raised 3 cm from the floor by inserting two 5 cm supporting screws at a 90° angle to one another 1 cm from the end of the tube. The burrow was filled with maximum weight of burrowing material (standard bedding substrate) and placed in a large holding cage that had 200 ml of standard bedding substrate. A single mouse was placed in the cage and the experiment was conducted for 1 hour in light. After the experiment the amount of burrowing material left in the tube was measured. The researcher was present in the room while the experiment was ongoing. Prior to the first day of testing, animals were trained twice to avoid neophobia.

**Neuropathology**

**Brain section preparation**

The brains were removed as described above, and immersion-fixed in 10% formalin for 2 days at 4°C with gentle shaking. Fixed brains were paraffinized with each cassette containing age-matched HD and control samples. Sections were cut to be 4 µm thick using a standard microtome with a cooled blade.

**Immunohistochemistry staining**

Tissue sections were deparaffinized by incubating slide 2x 5 min in xylene, 2x 5 min in 100% ethanol, 5 min in 95% ethanol, 5 min in 70% ethanol, 5 min in 50% ethanol, and 5 min in 1x PBS. Sections were then treated for antigen-retrieval by boiling slides for 20 min in 100°C citrate buffer (pH = 8) and then left to cool down at room temperature (RT) for 20 min. For staining of NIIs, sections were treated with 25 μg/ml proteinase K for 30 min at 37°C to remove cytosolic Htt while retaining the NIIs. The tissue sections were then treated with 0.3% H_2_O_2_ for 30 min at RT to inactivate endogenous peroxidases. Sections were permeabilized with blocking buffer (2.5% normal horse serum in 1x PBS) for 1 h at RT and then incubated with primary antibodies in blocking buffer for 30 min at RT followed by 2 x 5 min washes in 1x PBS. After that, sections were incubated with secondary antibodies in blocking buffer for 30 min at RT, followed by 2 x 5 min 1x PBS washes and colorized with NovaRED substrate (SK-4800, Vector Laboratories, Burlingame, CA). Slides were mounted with Vectashield Vibrance antifade mounting medium (Vector Laboratories). Primary antibodies used: Huntingtin 1:100 (ab109115, Abcam, Cambridge, UK); GFAP 1: 500 (GA524, Dako Omnis); Iba1 1: 200 (019-10741, Wako Chemicals).

**Immunofluorescence staining**

Deparaffinization and antigen retrieval were performed as described above. Following antigen retrieval, sections were permeabilized with blocking buffer for 1 hour at RT and then incubated with primary antibodies in blocking buffer for 30 min at RT. Sections were wash twice in 1x PBS for 5 min and incubated with secondary antibody in blocking buffer for 30 min at RT, followed by 2 x 5 min PBS washes. Slides were mounted with Vectashield Vibrance antifade mounting medium (Vector Laboratories). Primary antibody: huntingtin 1:100 (ab109115, Abcam, Cambridge, UK). Secondary antibody: Alexa Fluor 488 donkey anti-rabbit 1:250 (Jackson ImmunoReseach, RRID: AB_2313584).

**In situ hybridization**

In situ hybridization was performed using a RNAscope 2.5 HD Assay RED kit (ACD BioTechne; Newark, CA) according to supplier protocol. In brief, tissue sections were deparaffinized by submerging 2x for 5 min in xylene, then 2x for 1 min in 100% ethanol. Sections were then treated with H_2_O_2_ for 10 min at RT for endogenous peroxidase inactivation. Afterwards, slides were placed in target retrieval buffer (ACD BioTechne) for 15 min at 100°C followed by washing in nuclease-free water for 30 sec at 100°C. Sections were left to airdry for 5 min at RT before treatment with RNAScope Protease Plus (ACD BioTechne) for 30 min at 40°C. Slides were then incubated with probes for 2 hours at 40°C. Slides were then incubated with serial RNAScope probe-amplification reagents at 40°C: AMP1 for 30 min, AMP2 for 15 min, AMP3 for 30 min, AMP4 for 15 min; at RT: AMP5 for 30 min and AMP6 for 15 min. Slides were colorized by RNAScope Fast-RED for 10 min at RT. Autofluorescence quenching was performed for 5 min with TrueView (Vector Laboratories) according to supplier protocol, followd by counterstaining with DAPI (1:20000 in 1x PBS with 2.5% normal horse serum). Slides were mounted with Vectashield Vibrance antifade mounting medium (Vector Laboratories). Used probes: Mm-Ccnd1: RNAscope Probe 442671; Lot: 212848; Mm-Htt: RNAscope Probe 405881; Lot: 172268; Mm-Ppp1r1b: RNAscope Probe 405901; Lot: 172778.

**Quantification of huntingtin aggregation, RNA expression and colocalization**

Images of sections were taken with a Zeiss Axio A1/D1 microscope (Zeiss, Oberkochen, Germany. Images of *Htt* mRNA expression were deconvoluted with Huygens software (Scientific Volume Imaging (SVI), Hilversum, Netherlands) and particle counting of Htt^+^ aggregates, *Htt* mRNA, and colocalization of Htt aggregates with nuclei was performed with IMARIS software using default settings (Bitplane, Oxford Instrument, England). Images of *Ppp1r1b* mRNA expression were processed and analyzed using FIJI [10]. to measure integrated density in each image.

**Visualization and Quantification of *Ccnd1* mRNA ISH staining**

For quantification of *Ccnd1* mRNA ISH-staining in the anterior cerebellum of 9-month-old mice, 2x2 tile image stacks (16 bit) of lobule IV/V (white square in Fig. 4 B) were taken on a Zeiss LSM700 confocal microscope with 488 nm and 555 nm lasers using a 20x objective (Plan-Apochromat 20x/0.8 M27). We analyzed five cassettes, each containing HD and control brains, resulting in five matched biological replicates per genotype. To image sections from the same cassette the master gain and offset were adjusted for the section with the highest Ccnd1 signal intensity and the same settings were applied to all sections on the slide. Ccnd1 signal in the granular layer was quantified using FIJI. Stacked images were projected for by sum calculation and background was subtracted using a rolling ball algorithm (radius: 50 px). Regions of interest (ROI) were defined by using the polygon tool to outline the granular layer based on DAPI staining. The mean fluorescence intensity in ROIs was measured in the Ccnd1 channel, resulting in two measurements (right and left of arbor vita) for each image. As the control group failed the assumption of normal distribution (Shapiro-Wilk test W = 0.83, p = 0.03), intensity in HD and Control stainings was compared using a Wilcoxon rank-sum test, designed for non-parametric, matched samples. Since we found that age of the paraffine blocks and time between sectioning and staining greatly influenced the overall staining quality, we found it appropriate to treat HD and Control sections which originating from the same paraffine blocks, and were therefore processed, stained, and scanned under same conditions, as paired samples.

For visualization in Figure S7, images of cerebellar lobules III-V were taken from HD and Control sections originating from the same cassette. Using FIJI, images were converted to 8-bit and a scale bar of 250 microns was added (pixel size = 0.1633096 micron). Image brightness and contrast for each channel was adjusted first for the HD section, as it had the higher signal intensity, and then the same minimum and maximum displayed values were applied to the control section.

**RiboTag RNAseq**

**Immunoprecipitation of cell type specific RNA with RiboTag**

Tissue homogenates were prepared from deep frozen samples in Polysome buffer (PSB) containing 50 mM Tris (pH 7.5), 100 mM KCl, 12 mM MgCl_2_, 1% IPEGAL CA-630, 1 mM DTT, 60 U/ml RiboLock RNase inhibitor (ThermoScientific, Waltham MA), 100 µg/ml cycloheximide (Sigma-Aldrich, St. Louis MO), and 2x SigmaFast EDTA-free protease inhibitor cocktail (Sigma-Aldrich, St. Louis MO), using 200 µl ice-cold PSB per 0.01 g tissue. Homogenates were obtained using Wheaton Potter-Elvehjem homogenizers and PTFE pestles (DWK Life Science, Millville, NJ) with a motorized homogenizer (HEI-Torque Core, heidolph, Schwabach, Germany) at 450 rpm, then centrifuged at 10000 xg for 10 min to obtain supernatant (S1). Total RNA was isolated from 200 µl S1 by phenol-chloroform extraction using Trizol, and purified using the Qiagen RNEasy kit (Qiagen, Hilden, Germany). Total RNA was eluted with 30 µl nuclease-free water and stored at -72 °C. The 900 µl of the remaining S1 were used for RiboTag immunoprecipitations. The S1 was pre-cleared using 75 µl protein-G dynabeads (PGDB; Invitrogen, Waltham MA, Cat. 1009D, Lot: 00729875), conjugated with 9 µg IgG2b Isotype antibody (Invitrogen, Waltham MA; Cat. 14473285, Lot: 2025721). Beads were collected in a magnetic rack and cleared S1 was incubated with 36 µl anti-HA 12CA5 monoclonal antibody (Roche, Cat. 11666606001, Lot: 39746400), rotating at 4 °C for 90 min. 90 µl washed PGDB were resuspended in the S1-antibody mix and incubated rotating at 4 °C for 45 min. Beads were washed twice with 900 µl PSB, thrice with 900 µl HSB (containing 50 mM Tris (pH 7.5), 300 mM KCl, 12 mM MgCl_2_, 1% IPEGAL CA-630, 1 mM DTT, 20 U/ml RiboLock RNase inhibitor, 100 µg/ml cycloheximide, and 0.5x SigmaFast EDTA-free protease inhibitor cocktail) and once with 900 µl EHSB (as HSB, containing additional 300 mM NaCl) by careful resuspension in wash buffer and incubation at 300 rpm, room temperature, 5 min. Following washing, ribosomes and mRNA extracted using phenol-chloroform extraction with Qiazol (Qiagen, Hilden, Germany) and clean up using the Qiagen RNeasy Mini kit according to protocol. Columns were eluted with 30 µl of nuclease-free water and stored at -72 °C. To increase yield for PV samples, duplicated IPs were performed using two aliquots of 900 µl S1 were taken from same brain homogenate and pooled at the clean-up step.

**Library preparation and sequencing:** Libraries were prepared at SNP&SEQ Technology platform at NGI Uppsala, Sweden, using the Illumina TruSeq Stranded mRNA kit. Quality control and quantification of RNA samples and libraries was performed using Agilent Tapestation (Agilent, Santa Clara CA). Paired end sequencing (100 bp) was performed on an Illumina NovaSeq6000 sequencer using a S4 flow cell (Illumina, San Diego CA). Libraries of three samples failed initial sequencing and were re-sequenced at 150 bp PE and the same platform (indicated in metadata file as seq_run = 2).

**Alignment** was performed using the nf-core/rnaseq 3.0 analysis pipeline[11] using default settings. STAR and Salmon were used for alignment and quantification. Sequences for ERCC spike ins and RiboTag-HA tag were included as additional Fasta file. Samples were kept if they contained >30M mapped reads and <20% ribosomal RNA reads.

**Bioinformatic analysis**

**Principle component analysis** was done by calculating the variance for protein-coding genes based on log-scaled transcripts per million (TPM) values across either RiboTag IP and total RNA input samples. Top 500 most variable genes were used for principal component analysis with prcomp() and visualized with ggplot2 (v 3.3.3). To analyze enrichment of cell type-specific marker genes in RiboTag IP samples we normalized log2-transformed TPM values to total RNA and calculated the row-wise, normalized z-score using the formula: Z = (x – mean(total RNA))/SD(row), where x = sample TPM and SD = row-wise standard deviation. Heatmaps were visualized using pheatmap (v 1.0.12).

**Differential expression analysis** was performed with DESeq2 (v 1.30.1) to compare disease and control samples for each cell type. Genes were prefiltered to include only protein coding genes with a row-wise mean count > 10. Genes with a false discovery rate (FDR)-adjusted p-value ≤ 0.05 were considered as differentially expressed.

**Overrepresentation analysis** (ORA) for differentially expressed genes was performed for each investigated cell type using the enrichGO() function from clusterProfiler (v 3.18.1) for Biological Processes, with a cutoff of FDR < 0.05. Bulk RNA data for cerebrum and cerebellum in HD is available on hdinhd.org or at Gene Expression Omnibus under accession number (GEO:GSE65776)[12]. Bulk data for SCA1 can be found under accession number (GEO:GSE122099).

**Gene set enrichment analysis (GSEA)** for GO Biological Process (c5.go.bp.v7.4.symbols; gsea-msigdb.org) and KEGG pathways (KEGG_mouse_2019; maayanlab.cloud/Enrichr) was performed using piano (v 2.6.0). Gene set statistics for different directionality classes were calculated with the piano R package[13] using six different methods to calculate statistical significance by setting argument for “geneSetStats” to “mean”, “median”, “sum”, “stouffer”, “reporter” or “tailStrength”. Median consensus scores were calculated based on adjusted p-values using the integrated consensusScores() function. Terms with distinct directional adjusted p values ≤ 0.05 in at least two of the six applied gene set statistics were included in result. For visualization, GO terms were collapsed to parent terms using rrvigo (v 1.2.0) by semantic similarity (“Resnik”, threshold = 0.8).

**Chip-X enrichment analysis (ChEA)** was performed according to Lachman et al.[14] using the enrichr R-package (v ) to analyze overrepresentation of DEGs among gene sets defined in the “ChEA 2016” collection of transcription factor-associated genes based on various ChIP (chromatin immunoprecipitation) methods.

**Overlap analysis** of cell type-specific DEGs from RiboTag IPs with RNAseq data was calculated using Fisher’s exact test with the GeneOverlap (v 1.26.0) R package, using the average number of protein coding genes detected in IP samples (12519) as background.

**References**

1. Lin CH, Tallaksen-Greene S, Chien WM, Cearley JA, Jackson WS, Crouse AB, et al. Neurological abnormalities in a knock-in mouse model of Huntington’s disease. Hum Mol Genet [Internet]. 2001;10:137–44.

2. Heng MY, Duong DK, Albin RL, Tallaksen-Greene SJ, Hunter JM, Lesort MJ, et al. Early autophagic response in a novel knock-in model of Huntington disease. Hum Mol Genet. 2010;19:3702–20.

3. Kaczmarczyk L, Reichenbach N, Blank N, Jonson M, Dittrich L, Petzold GC, et al. Slc1a3-2A-CreERT2 mice reveal unique features of Bergmann glia and augment a growing collection of Cre drivers and effectors in the 129S4 genetic background . Sci Rep. 2021/03/08. 2021;11:5412.

4. Kaczmarczyk L, Schleif M, Dittrich L, Williams RH, Bansal V, Rajput A, et al. Distinct translatome changes in specific neural populations precede electroencephalographic changes in prion- infected mice. 2022;1–29.

5. Vong L, Ye C, Yang Z, Choi B, Chua S, Lowell BB. Leptin Action on GABAergic Neurons Prevents Obesity and Reduces Inhibitory Tone to POMC Neurons. Neuron [Internet]. Elsevier Inc.; 2011;71:142–54. Available from: http://dx.doi.org/10.1016/j.neuron.2011.05.028

6. Haimon Z, Volaski A, Orthgiess J, Boura-Halfon S, Varol D, Shemer A, et al. Re-evaluating microglia expression profiles using RiboTag and cell isolation strategies. Nat Immunol. 2018;19:636–44.

7. Hippenmeyer S, Vrieseling E, Sigrist M, Portmann T, Laengle C, Ladle DR, et al. A developmental switch in the response of DRG neurons to ETS transcription factor signaling. PLoS Biol [Internet]. 2005/04/20. 2005;3:e159. Available from: https://www.ncbi.nlm.nih.gov/pubmed/15836427

8. Dittrich L, Petese A, Jackson WS. The natural Disc1-deletion present in several inbred mouse strains does not affect sleep. Sci Rep [Internet]. 2017/07/20. 2017;7:5665. Available from: https://www.ncbi.nlm.nih.gov/pubmed/28720848

9. Steele AD, Jackson WS, King OD, Lindquist S. The power of automated high-resolution behavior analysis revealed by its application to mouse models of Huntington’s and prion diseases. Proc Natl Acad Sci U S A [Internet]. 2007;104:1983–8. Available from: https://www.ncbi.nlm.nih.gov/pubmed/17261803

10. Schindelin J, Arganda-Carreras I, Frise E, Kaynig V, Longair M, Pietzsch T, et al. Fiji: An open-source platform for biological-image analysis. Nat Methods. 2012;9:676–82.

11. Ewels PA, Peltzer A, Fillinger S, Patel H, Alneberg J, Wilm A, et al. The nf-core framework for community-curated bioinformatics pipelines. Nat. Biotechnol. United States; 2020. p. 276–8.

12. Driessen TM, Lee PJ, Lim J. Molecular pathway analysis towards understanding tissue vulnerability in spinocerebellar ataxia type 1. Elife. 2018;7:1–32.

13. Väremo L, Nielsen J, Nookaew I. Enriching the gene set analysis of genome-wide data by incorporating directionality of gene expression and combining statistical hypotheses and methods. Nucleic Acids Res [Internet]. 2013;41:4378–91. Available from: https://doi.org/10.1093/nar/gkt111

14. Lachmann A, Xu H, Krishnan J, Berger SI, Mazloom AR, Ma’ayan A. ChEA: Transcription factor regulation inferred from integrating genome-wide ChIP-X experiments. Bioinformatics. 2010;26:2438–44.
